# RNA-Seq based genome-wide analysis reveals loss of inter-chromosomal regulation in breast cancer

**DOI:** 10.1038/s41598-017-01314-1

**Published:** 2017-05-11

**Authors:** Jesús Espinal-Enríquez, Cristóbal Fresno, Guillermo Anda-Jáuregui, Enrique Hernández-Lemus

**Affiliations:** 1 Computational Genomics Division, National Institute of Genomic Medicine (INMEGEN), 14610 Mexico City, Mexico; 2 Centro de Ciencias de la Complejidad, Universidad Nacional Autónoma de México (UNAM), 04510 Mexico City, Mexico; 3 UA AREA CS. AGR. ING. BIO Y S, CONICET - Universidad Católica de Córdoba, Córdoba, Argentina; 4 Department of Biomedical Sciences, School of Medicine and Health Sciences, University of North Dakota, 501 North Columbia Rd Stop 9061, Grand Forks, ND 58203 USA

## Abstract

Breast cancer is a complex heterogeneous disease. Common hallmark features of cancer can be found. Their origin may be traced back to their intricate relationships governing regulatory programs during the development of this disease. To unveil distinctive features of the transcriptional regulation program in breast cancer, a pipeline for RNA-seq analysis in 780 breast cancer and 101 healthy breast samples, at gene expression and network level, was implemented. Inter-chromosomal relationships between genes resulted strikingly scarce in a cancer network, in comparison to its healthy counterpart. We suggest that inter-chromosomal regulation loss may be a novel feature in breast cancer. Additional evidence was obtained by independent validation in microarray and Hi-C data as well as supplementary computational analyses. Functional analysis showed upregulation in processes related to cell cycle and division; while migration, adhesion and cell-to-cell communication, were downregulated. Both the BRCA1 DNA repairing signalling and the Estrogen-mediated G1/S phase entry pathways were found upregulated. In addition, a synergistic underexpression of the *γ*-protocadherin complex, located at Chr5q31 is also shown. This region has previously been reported to be hypermethylated in breast cancer. These findings altogether provide further evidence for the central role of transcriptional regulatory programs in shaping malignant phenotypes.

## Introduction

Breast cancer is a complex disease. This heterogeneous pathology is characterized by an intricate interplay between different biological aspects such as DNA genomic alterations, gene expression deregulation, hormone disruption, metabolic abnormalities, protein failure, signalling pathway alterations and also environmental determinants. These aspects in turn influence the onset, development, outcome of breast carcinomas as well as the appearance of metastases^1^. The heterogeneity of breast cancer can be observed at the molecular, histological, and functional level, all of which have clinical implications^2, 3^. However, most breast cancer manifestations exhibit shared features, such as upregulation of the cell cycle, cell cycle checkpoints evasion^4, 5^, inflammatory responses^6–8^, immune response evasion^9, 10^ and deregulation of the genetic expression^11, 12^ among others.

Breast cancer is one of the most studied diseases. However, we still have not a complete, integrative understanding of the role transcriptional regulation establishes and modifies the cancer cellular landscape: particularly, how the regulatory program of a “healthy” cell drifts towards a “cancerous” phenotype. In this context, high-throughput omic technologies have provided us unprecedented tools to study the alterations found in cancer at a deeper level. They have become essential instruments for both basic and clinical research, to fathom the multi-layered relationships between the actors that participate in this complex disease. However, relatively small sample sizes have not enabled the construction of a complete portrait of the disease.

In an attempt to bring light to the understanding of the cell regulatory program during cancer, we set to study a comprehensive collection of breast invasive carcinoma RNA-Seq samples from The Cancer Genome Atlas (TCGA)^13, 14^. State-of-the-art computational methodologies were used for quality control and data preprocessing/processing. Differential gene expression and diverse functional enrichment procedures were applied, to observe the main differences between the two phenotypes.

To unveil how the transcriptional regulatory program is composed in healthy and cancerous samples, we constructed gene regulatory networks (GRNs) where the nodes correspond to genes and the links that connect them represent a statistical dependence. In this context, these dependencies can be understood as correlations in transcriptional regulation processes.

GRNs may actually refer to several types of gene networks. In general, it may include the term “transcriptional regulatory network” (TRN) which is used to describe directed networks, that may take into account transcription factor/promoter affinity obtained from transcription factor binding site analyses, as well as sequence-capture experimental data. The term GRN may also refer to undirected networks like those obtained from probabilistic modeling (using either correlation or mutual information measures) that reflect co-regulation and co-expression patterns, and (unlike TRNs) are able to capture indirect interactions, not caused by direct physical contacts. Such gene regulatory networks (like the ones we calculated in this work) are, in general not causal but probabilistic.

Gene regulatory networks of the cancerous and non-cancerous mammary tissue samples were inferred, constructed, analysed and compared. The large number of samples of both cases and controls allowed us to construct whole-genome networks with high statistical significance. We found that almost every strong relationship in the cancerous network occurs between genes belonging to the same chromosome, with few relationships across chromosomes, but more importantly, these intra-chromosomal links occur between genes located at chromosomal neighbouring regions. These intra-chromosomal *clusters* present a consistent differential expression pattern: either overexpressed or underexpressed. Meanwhile, the healthy network presents several relationships between genes from different chromosomes, as well as intra-chromosomal correlations. We argue that this is a strong evidence of a new feature in breast cancer: loss of long-range transcriptional regulation. This observation is consistent with recent Hi-C data obtained from MCF7 and MCF10a breast cancer cell lines^15^, and suggests the need for further experimental analysis of this phenomenon. Our approach tries to capture common features of breast cancer, such as processes and genome-wide relationships that are altered in disease, which may help us to understand the transcriptional regulation present in the development of this complex pathology.

## Results

### Mutual information networks reveal evident structural differences between cancer and controls

**Figure 1:**
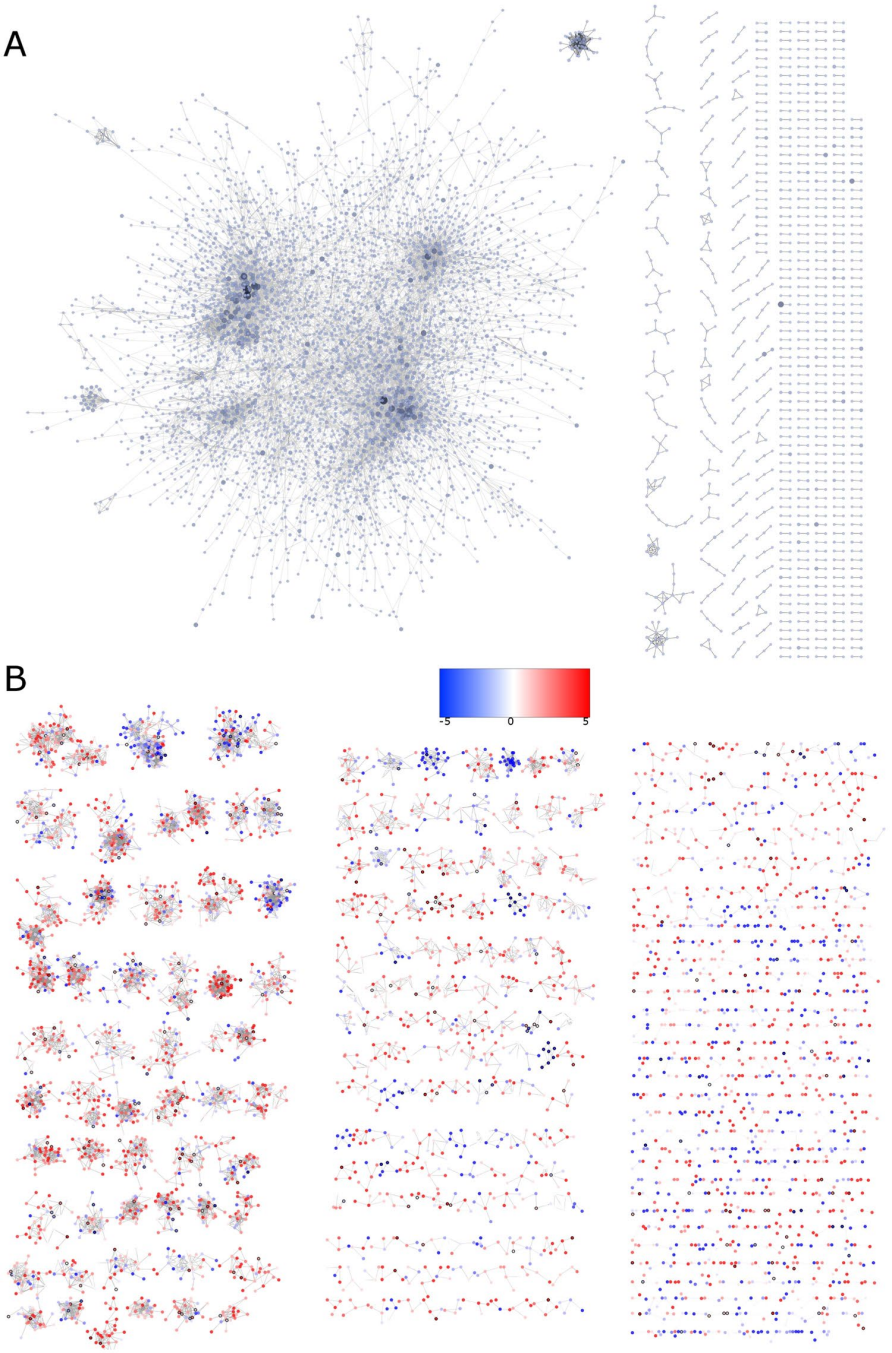
Healthy and cancerous mutual information inferred networks. This figure shows the architectural features of each network. (**A**) Healthy network (HN) where the higher color intensity, the higher the vertex degree is. (**B**) Cancerous network (CN) where red/blue vertices represent over/underexpressed genes. Notice the presence of a large, dominant component in the HN, which is clearly not the case for the CN, where several small components coexist. It is also observable the predominance of overexpressed (red) or underexpressed (blue) clusters in CN.

To unveil how the transcriptional regulatory program is composed in healthy and cancerous samples, independent mutual information (MI) based gene regulatory networks were constructed, using 780 breast invasive carcinoma and 101 healthy RNA-Seq samples from The Cancer Genome Atlas^13^ (see Material and Methods section and Supplementary Table S1). In the network, vertices correspond to genes and the edges that connect them represent the MI between genes, which can be understood as correlations in transcriptional regulation processes. By looking at the network’s topology for both healthy and cancerous networks (Fig. 1), it can be seen that the architecture is completely different, despite the fact that both networks were created using the same visualization algorithm, i. e., Cytoscape’s profuse force-directed layout. The healthy network (HN, Fig. 1A) contains a giant connected component depicted by the color degree intensity of their vertices. On the contrary, the cancer network (CN, Fig. 1B) has several small disconnected components, where red/blue vertices represents over/underexpressed genes. Notice that each connected component in the CN is predominantly overexpressed or underexpressed, suggesting a common regulatory process for the whole component.

As it can be argued from Fig. 1, global network parameters also differ between HN and CN. Table 1 shows the principal measures for both networks. In particular, network diameter and connected components reflect the strong differences between HN and CN, where the giant component of the HN determines the network structure. Regarding gene parameters, degree of CN genes is in general smaller than HN (Table 2, see also Supplementary Tables S2 and S3); that is expected since the largest component in CN contains only 134 genes, meanwhile the giant component in HN has 4,214 out of 5,395.

**Table 1:** Network parameters for Healthy (HN) and Cancerous (CN) phenotypes.

Parameter	HN	CN
Connected Components	478	842
Largest Component	4,214	134
Diameter	22	14
Connected Pairs	17,757,588	162,552
Avg. Shortest Paht Length	6.727	3.428
Avg. Degree	4.319	4.64
Genes	5,395	5,022
Density	8.01 × 10^−4^	9.24 × 10^−4^
Clustering Coefficient	0.135	0.377

Notice that the network diameter as well as the connected pairs (100×) are considerably larger in HN with respect to CN. It is opposite to the case for the number of connected components.

**Table 2:** Top 10 vertex degrees for Healthy (HN) and Cancerous (CN) networks.

HN gene	Degree	CN gene	Degree
ZBTB21	98	NEURL4	52
FAM160A1	92	SLC25A11	50
PLK3	82	DPH1	50
TSC22D2	72	PSMB6	48
ACVR2B	67	ANKFY1	48
FKBP2	64	TSR1	44
HOOK1	61	C1QBP	43
SPATA2L	59	RPA1	42
AP1M2	55	HSF1	41
SLC25A25	52	ZNF7	40

Notice that the more connected gene in CN (NEURL4) has the same number of neighbours than the tenth highest degree node in HN (SLC25A25).

#### Cancerous networks show loss of inter-chromosomal regulation

**Figure 2:**
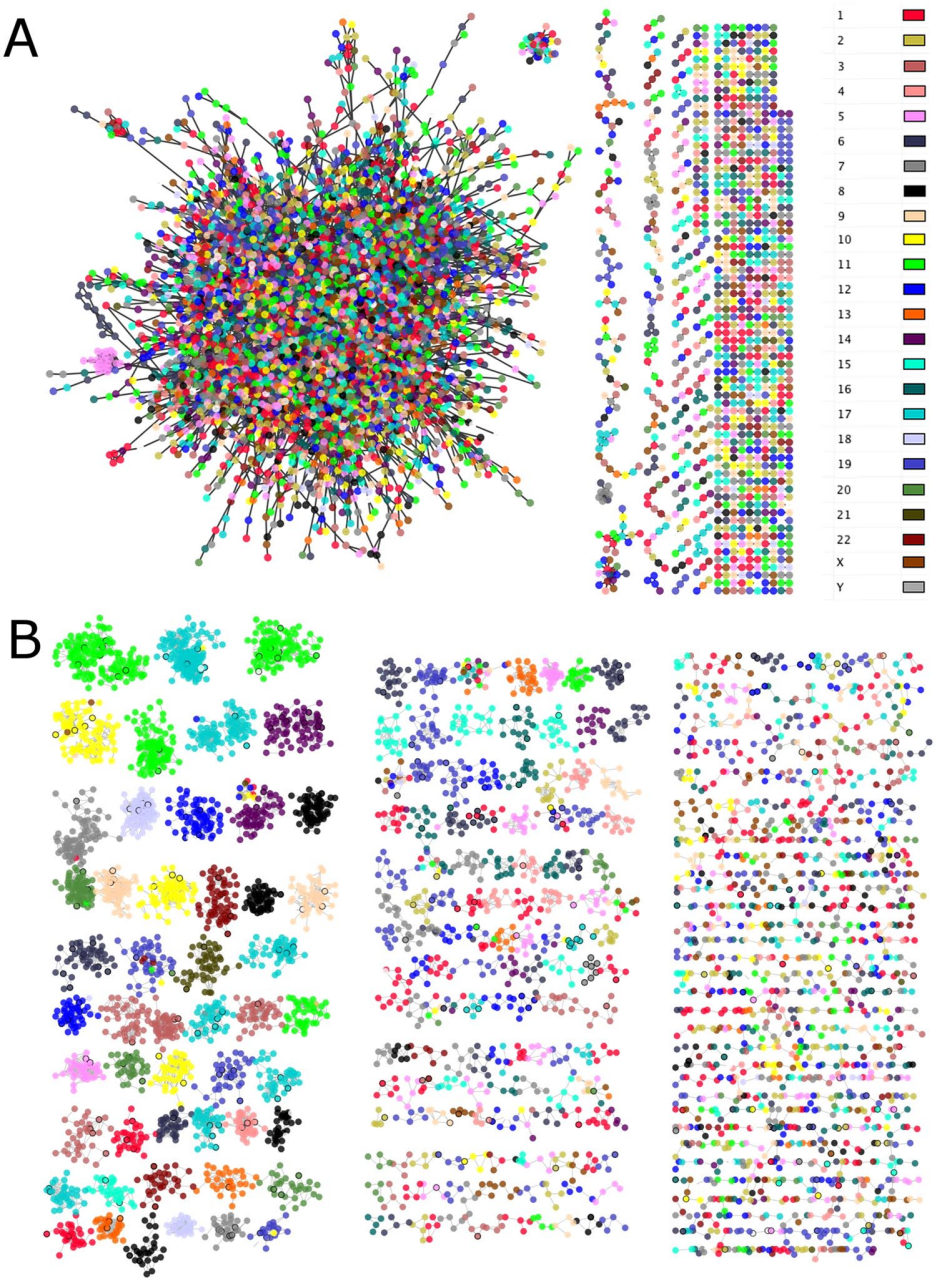
Inter-chromosomal regulation loss in cancerous network. (**A**) Healthy network (HN). (**B**) Cancerous network (CN). This figure shows the architectural features of each network. Both networks are depicted with the same layout as in Fig. 1. The color code is according to the chromosome location in which each gene is placed. Notice the presence of a single giant component in the HN, which is not the case for CN, where several small components coexist. Furthermore, in the HN of panel (A) all genes in the giant component belong to different chromosomes, meanwhile in the CN of panel (B), almost all components are composed of genes which belong to the same chromosome.

Given the aforementioned result on the predominance of overexpressed or underexpressed clusters in CN, we inspected the chromosomal location of all genes in the both networks, to observe whether the distribution of chromosomal location of genes in CN is compartmentalised. If this statement holds, it would indicate that possibly the transcriptional regulation in cancer occurs preferentially in neighbouring regions. The results can be observed in Fig. 2, where the genes of both networks presented in Fig. 1 are now coloured by the chromosome to which they belong to. Surprisingly, the majority of the genes for each component present in the CN belongs to the same chromosome (Fig. 2B). This is not the case of the giant component in the HN (Fig. 2A), where genes are connected to other genes located on different chromosomes.

To further assure that this finding is not an artefact of the MI cut-off value used, as the networks presented in Figs 1 and 2 have been constructed using only the top 0.01% (11,675 edges), networks with a less astringent MI cut-off value (0.1%) were constructed. This new cut-off value created networks with 116,503 edges and reaffirmed the consistency of the obtained results (see Supplementary Figure S1), i. e., the transcriptional regulation takes place in the same chromosome in the CN, while in the HN, genes regulate other genes from different chromosomes. In parallel, we constructed other two networks using the top 0.001% MI values (1,168 edges), in order to observe whether or not the HN giant component is still connecting groups of genes from different chromosomes. The results showed a pattern of neighbours that behave alike Fig. 2 (Supplementary Figure S2): in the HN, genes are connected to other genes of different chromosomes, while the CN components only have connected genes that belong to the same chromosome.

**Figure 3:**
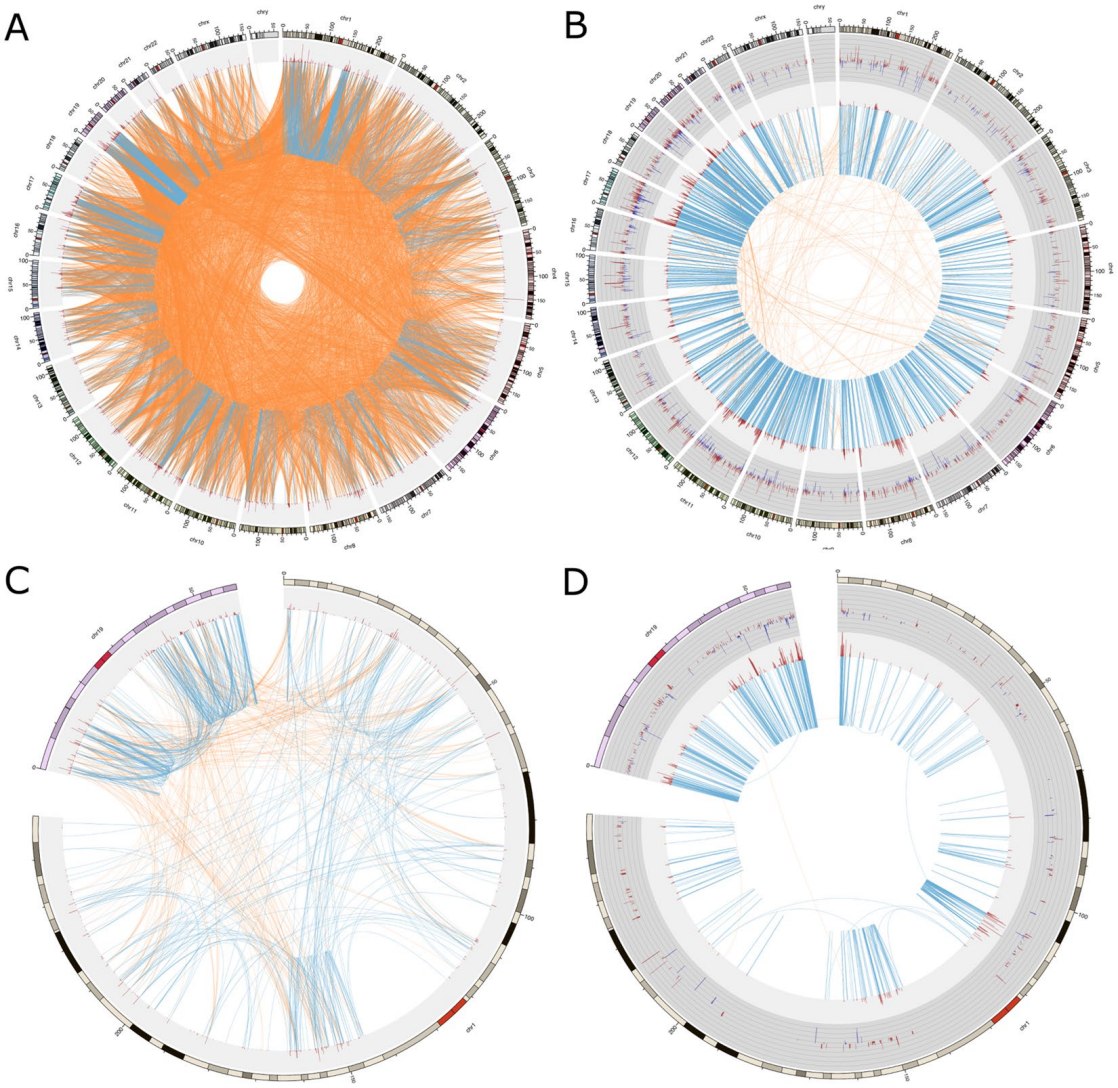
Inter-chromosomal regulation loss. The circos plots show the dramatic difference in the connectivity of healthy network (HN) and cancerous network (CN). (**A**) The HN using the top 0.01% mutual information (MI) values. The blue lines represent gene intra-chromosomal relationships. Orange lines join inter-chromosomal genes. The next outer circle (grey) represents the degree of each gene (number of neighbours of each gene) as red peaks height proportional to its degree. The external circle indicates the chromosomal location where regions are separated by squares. Notice in panel (A) the high density of the inter-chromosomal relationships (around 11,000 edges). On the contrary, in panel (B) the CN has a high density of intra-chromosome relationships, meanwhile, the inter-chromosome links are almost absent. It is worth to mention that both networks contain the same number of links. In panel (B) it is also depicted in a dark-grey circle, the differential expression of its genes: blue/red histograms indicate under/overexpression respectively. The size of the line is proportional to the differential expression value. Panels (C and D) are a zoom-in for HN (panel C) and CN (panel D) of chromosomes 1 and 19, which shows a remarkable difference in the chromosomal distance between edges. In panel (C) genes are linked to Chr19 but are not close between them, meanwhile, for panel (D) the majority gene relationships takes place within its neighbourhood.

To further explore gene relationships circos plots were constructed using gene chromosomal locations for the HN (Fig. 3A) and the CN (Fig. 3B). Clearly, the majority of edges present in the HN connects genes from one chromosome to any other. On the other hand, the majority of connections in the CN is given between genes on the same chromosome. Figure 3C and D show a zoom-in only for chromosome 1 and 19 of Fig. 3A and B respectively, in order to give a detailed view of the distribution of the interactions. Despite the fact that the relationships are inside the chromosome 19, it can be observed once again that in the HN (Fig. 3C) they are distributed along the whole chromosome, meanwhile for the CN (Fig. 3D), the relationships occur very closely.

#### The *γ*-protocadherin cluster may be involved in several downregulated processes in breast cancer

**Figure 4:**
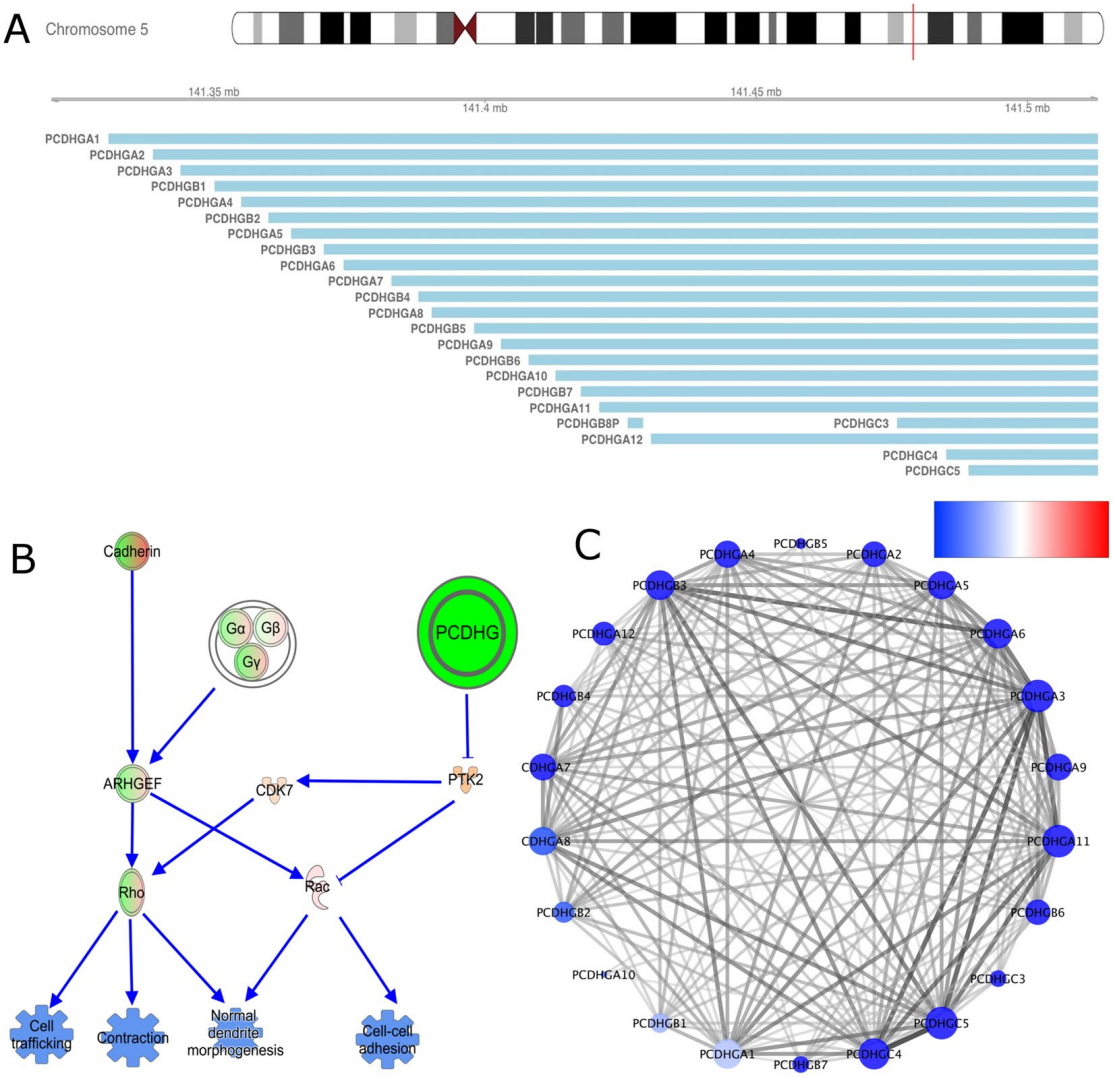
*γ*-Protocadherin complex (PCDHG) and its putative role in breast cancer. (**A**) Chromosomal localization of the 22 *γ*-protocadherins. (**B**) Signalling pathway involving G-proteins, protocadherins and their targets in the cell trafficking, contraction and cell adhesion processes. Molecule expression values are presented in colors and the expected activation state given by those expression values: under/overexpressed genes in green/red-orange respectively. Blue events represent downregulated processes. Red-green vertex represent molecule complexes that include overexpressed and underexpressed molecules together. (**C**) The PCDHG cluster obtained in the cancer network. Notice that all genes are underexpressed (blue color). Transparency and color edges intensity are according to the mutual information values obtained.

Both HN and CN network’s components were ranked by its network density. This parameter gives a clear idea of how interconnected a component is, because it can be thought as the number of existing edges in a network cluster divided by the total number of network edges. The most dense component of the CN is composed exclusively by 22 genes, which encode the 22 proteins in the *γ*-protocadherins cluster (PCDHG, Fig. 4) involved in the control of neuron development^16, 17^. All of the genes present in this cluster are underexpressed with respect to the HN and are located at 5q31 chromosomal region (Fig. 4A and C). This region has been found to be hypermethylated in breast cancer^18^.

Owing to this fact, we focused our attention on previous PCDHG complex activity reports on breast cancer. As far as the authors know only in Shima *et al*. work^19^ somatic mutations of PCDHGB4 gene were reported. However, there are a large number of publications in the literature regarding the effects that knockout and knockdown of PCDHGs may have in mouse neuron development. Neonate mice PCDHGs complex knock-out results in death^16, 17^, probably because apoptosis is highly active in knock-out mice. On the other hand, PCDHGs complex knockdown results in strong defects in dendrite development, control of actin dynamics, microtubule assembly/morphogenesis^16, 17^ and cell adhesion^20^. It has been observed that the PCDHG cluster controls the participation of Pyk2 (PTK2B), which in turn regulates Rac1 to promote a normal dendrite morphogenesis^20^ (Fig. 4B). The role that these processes may have in breast cancer is yet to be fully understood.

### Differential expression analysis

An expression analysis was carried out yielding a total of 1,431 differentially expressed genes (DEGs) between experimental conditions (see Supplementary Table S4). The top overexpressed genes were collagen type XVIII alpha 1 chain (COL18A1) and matrix metallopeptidase 11 (MMP11) which are related to invasion and migration processes in several cancer types^21–23^ (Table 3). The bottom underexpressed observed genes in our analysis were the carbonic anhydrase 4 (CA4), alcohol dehydrogenase 1B (ADH1B) and the vascular endothelial growth factor D (FIGF). The CA4 gene participates in several biological processes^24, 25^, however, its exact biological function is still unknown. The ADH1B gene is involved mainly in the catabolism of ethanol^26^. Finally, FIGF is a c-fos-induced growth factor gene involved in angiogenesis, lymphangiogenesis and endothelial cell growth^27^. The complete DEG list was used as input for the following functional analyses as described in the following sections.

**Table 3:** Top and bottom five differentially expressed genes.

Gene Symbol	Log_2_FC	p-value	False Discovery Rate	References In Breast Cancer
COL11A1	5.069	1.77 × 10^−212^	4.59 × 10^−210^	21–23
MMP11	4.147	1.14 × 10^−223^	4.59 × 10^−231^	67–69
KIF4A	3.905	0	0	70–72
GRM8	3.559	3.36 × 10^−133^	2.52 × 10^−131^	73
TPX2	3.284	1.07 × 10^−313^	5.45 × 10^−310^	74, 75
$\vdots$	$\vdots$	$\vdots$	$\vdots$	$\vdots$
PI16	−4.651	3.86 × 10^−117^	2.31 × 10^−115^	
CPA1	−4.868	5.19 × 10^−122^	3.35 × 10^−120^	76
FIGF	−5.165	2.24 × 10^−198^	4.51 × 10^−196^	27
ADH1B	−5.697	5.58 × 10^−144^	4.74 × 10^−142^	
CA4	−6.431	2.3 × 10^−192^	4.4 × 10^−190^	

Linear model raw p-values results were corrected for multiple comparisons using the False Discovery Rate method. Gene expression differences between cancerous and healthy samples are presented in *log*_2_ scale (*log*_2_ *FC*).

### Functional analysis

#### Functional pathway topology analysis

**Figure 5:**
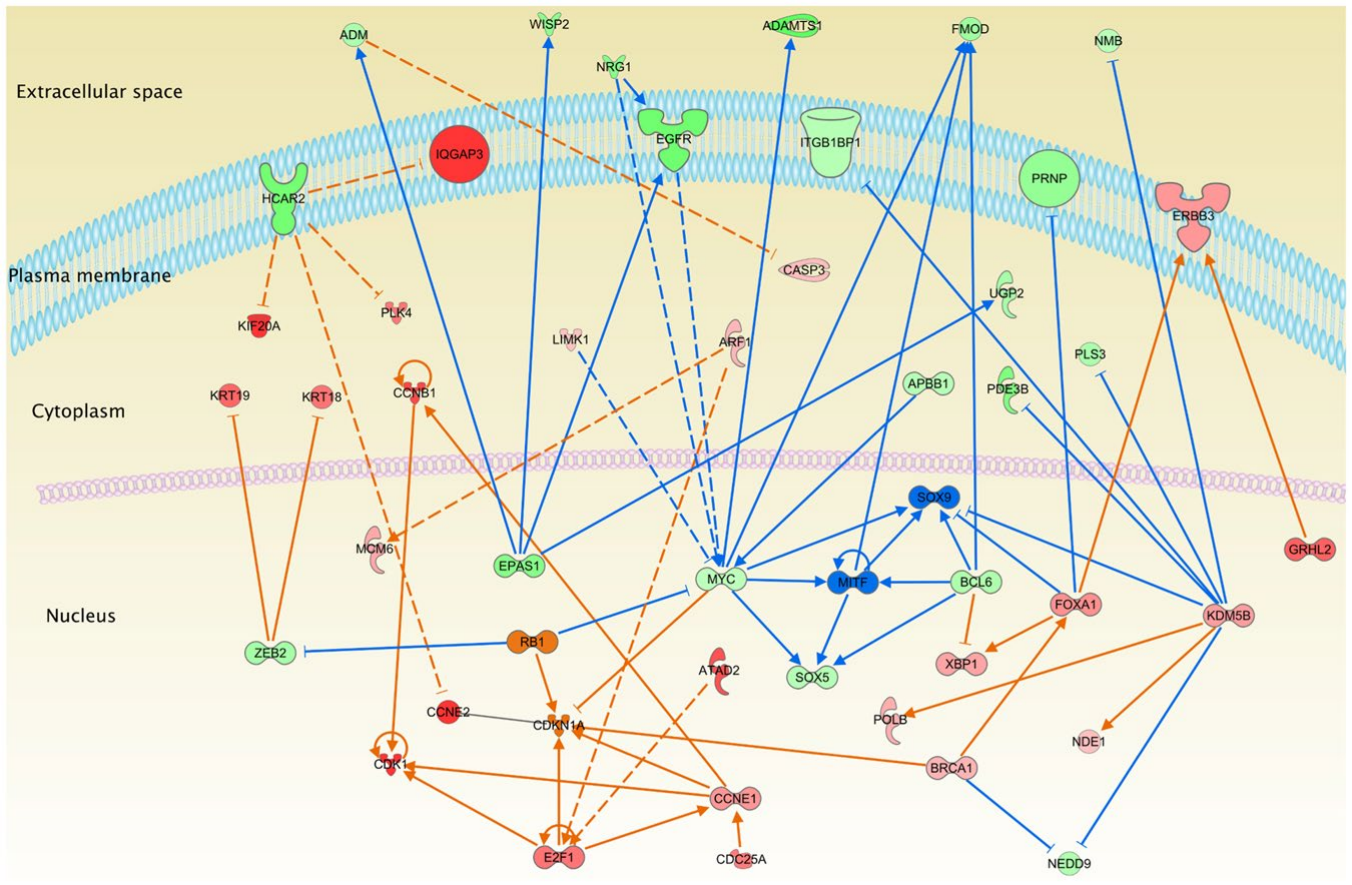
Ingenuity Pathway Analysis cancer network from RNA-Seq breast cancer samples. The molecules color represents the expression levels: green/red stands for under/overexpressed in cancer samples respectively. Blue/orange color indicates a predicted inhibition/activation of the molecule respectively. Blue and orange lines are depicted with the same color code. Molecules color intensity is presented in log_2_ FC scale. Notice that molecules in the extracellular space are underexpressed, whereas the inner components, in particular those that belong to the nucleus are overexpressed.

Causal networks were constructed using QIAGEN’s Ingenuity Pathway Analysis (IPA), in order to identify the main relationships involving the previously found DEGs. All the consistent-with-experiments relationships involved in cancer, according to IPA knowledge base (IKB), were chosen for the analysis. Results are depicted in Fig. 5 where green/blue elements represent underexpressed molecules, whereas red/orange represent its overexpressed counterpart. It can be observed that the outer elements are underexpressed in their majority, meanwhile, the inner components are mainly overexpressed. This configuration is a symptom of a high transcriptional activity. In addition, molecules outside the plasma membrane are related to extracellular matrix remodelling.

Our expression data were compared with IKB set of categories and biological functions. In this context, molecule expression levels present in any category were used to generate a significance activation Z-score for each process. Tables 4 and 5 presents the top and bottom five Diseases and Functions. It can be seen that the most increased functions (top of the table) were related to cell cycle, whereas blood vessel-related processes, growth, migration and differentiation were decreased. Complete results, including the molecules present in each category, are provided as Supplementary Table S5.

**Table 4:** Top five increased and decreased functions.

Annotated Functions	p-value	Activation Z-score	# Molecules
G2 phase	6.66 × 10^−05^	2.19	30
G2/M phase	1.31 × 10^−03^	2.186	23
Inflammation of organ	3.39 × 10^−04^	1.951	49
Synthesis of progesterone	1.47 × 10^−02^	1.399	7
Binding of endothelial cells	2.92 × 10^−03^	1.35	20
$\vdots$	$\vdots$	$\vdots$	$\vdots$
Vasculogenesis	1.74 × 10^−09^	−3.115	101
Angiogenesis	7.38 × 10^−10^	−3.176	103
Growth of epithelial tissue	4.12 × 10^−10^	−3.347	106
Migration of cells	1.08 × 10^−14^	−3.361	239
Differentiation of cells	2.40 × 10^−05^	−3.738	163

Note that the annotated functions are ordered using the activation Z-score. Companion p-values and the number (#) of molecules present in each function are also included.

**Table 5:** Top 5 comparison between inter-chromosomal network interactions and Hi-C experiments for healthy and cancerous data.

Phenotype	Chr	Symbol	GeneStart	Chr	Symbol	GeneStart	Network MI		Hi-C	
							Healthy	Cancerous	MCF10a	MCF7
Healthy	3	NR1D2	23986751	1	PRE3	7844380	0.4491	0.2441	1.112	0.5389
	22	TEF	41763337	1	PER3	7844380	0.4416	0.2576	0.8829	0.445
	8	ESRP1	95653302	1	RAB25	156030951	0.4381	0.058*	0.7815	0.9124
	21	ZBTB21	43406940	1	PLK3	45265897	0.4334	0.0154*	0.7083	0.6709
	3	NR1D2	23986751	1	UTS2	7903143	0.4252	0.1402*	1.112	0.5389
Cancerous	20	CSNK2A1	459116	11	CSNK2A3	11373489	0.150155*	0.601748	0.9135	1.214
	9	CBWD1	121041	2	CBWD2	114195268	0.247417	0.438327	0.9979	1.37
	18	KIAA1328	34409069	9	PGM5P2	69080240	0.127655*	0.436005	0.5589	0.8362
	3	ESRG	54666149	8	HHLA1	133073733	0.160935*	0.405771	0.8386	0.9733
	11	CSNK2A3	11373489	20	TBC1D20	416124	0.0338788*	0.30643	0.9135	1.214

*Not present at 0.01% MI cutoff.

**Figure 6:**
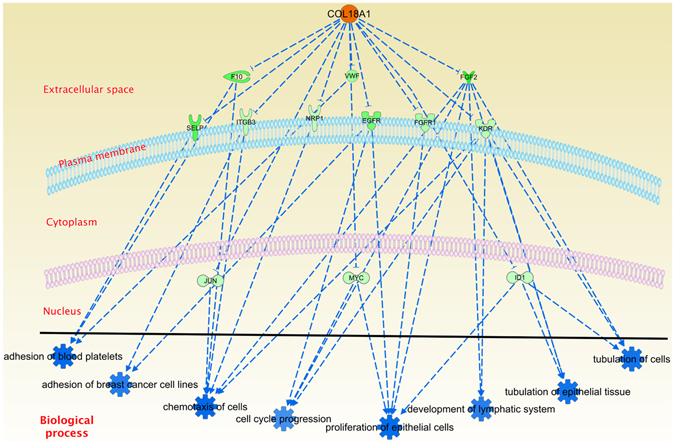
Upstream regulator analysis for COL18A1. COL18A1 overexpression (orange) decreases the expression of all molecules present in the figure (green), which accounts for the inhibition (dashed blue lines) of the biological processes indicated here.

IPA also provides a tool to predict the regulatory role that a molecule may have with respect to its known targets: the Upstream Regulator analysis. With this tool is possible to observe the biological functions that the regulatory targets have, giving an insight on the overall effect that such upstream regulator could exert. This task is performed by calculating a consistency score, which takes into account the expression levels of the regulator and their targets. In this work, we focused on collagenases since they have a crucial role in cell migration and extracellular matrix remodelling^28, 29^. Interestingly, COL18A1 gene had the highest consistency score, which was overexpressed in our cancerous samples. However, the regulatory role that this molecule has is mainly inhibitory. In addition, COL18A1 also decreases cell adhesion, chemotaxis, tubulation and cell cycle-related processes through some transcription factors as well as membrane proteins (Fig. 6).

There is similarity in the downregulated biological processes identified by the two independent analyses, i. e., the COL18A1 dependant decreased functions in Fig. 6, are similar to those found in the bottom five Diseases and Functions categories of Table 4. Given the fact that COL18A1 is not involved in any other enriched categories in this analysis, we can argue that these processes might be downregulated by more than one mechanism.

#### Canonical pathways analysis in breast cancer reveal cell cycle upregulation

**Figure 7:**
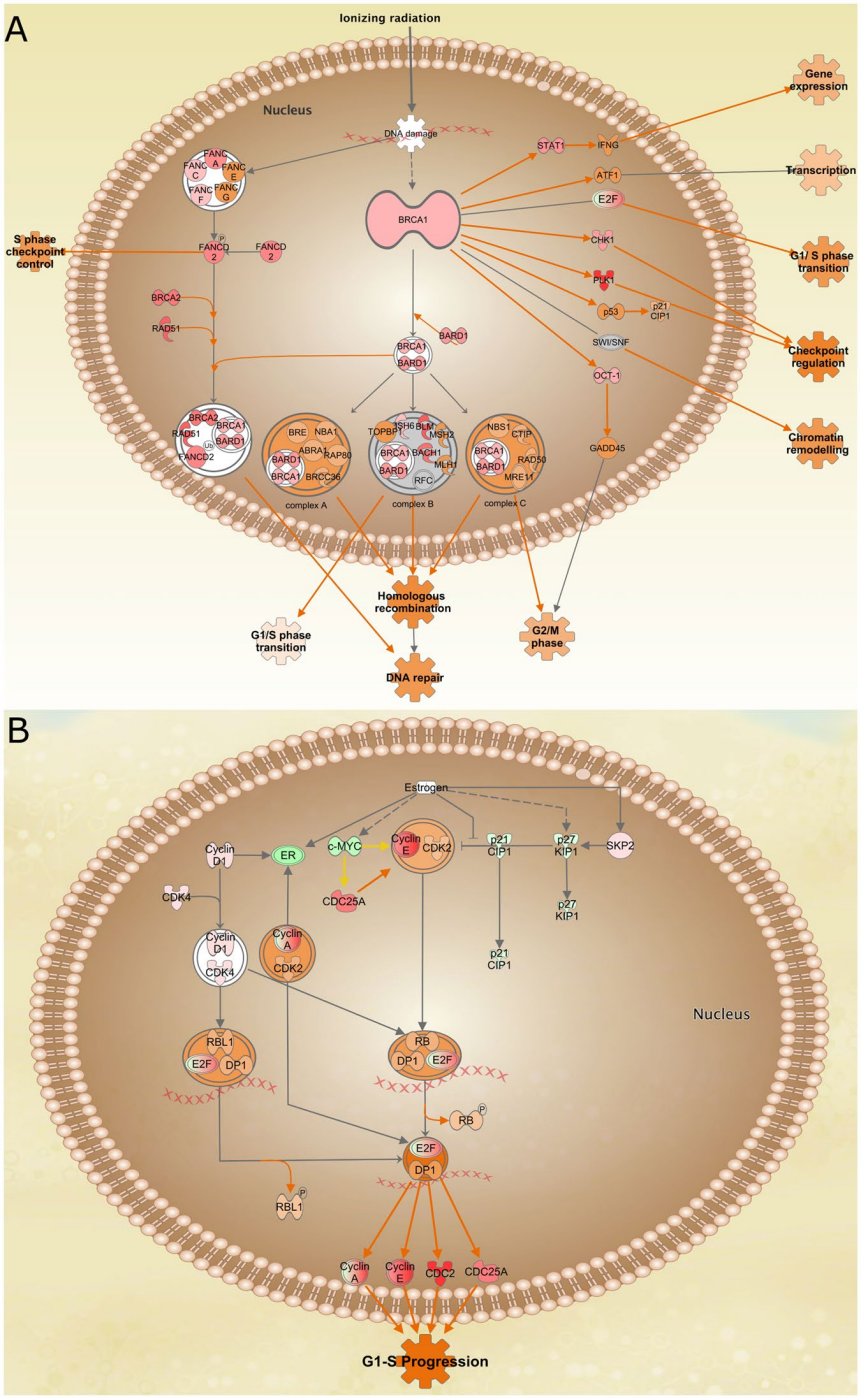
Top 2 activated canonical pathways. (**A**) Role of BRCA1 in DNA damage pathway. (**B**) Estrogen-mediated S-phase entry pathway. The molecules are depicted according to the standard color code of this manuscript. Notice that both pathways are consistent with cell cycle progression, a typical hallmark of cancer.

The Canonical Pathways curated in the IKB provided another analysis instance of our dataset, in which the molecules could participate in specific cellular events, i. e., sets of molecules interacting in a given pathway to perform a specific function according to their expression levels. The two most significant upregulated Canonical Pathways were the role of BRCA1 in DNA damage and the estrogen-mediated S-phase entry, which are related to cell cycle regulation processes. These pathways were overexpressed, since their molecules presented an expression value which is consistent with the expected values that the pathway would have, if said pathways were activated (Fig. 7A and B). It is worth mentioning that these upregulated pathways depend on two of the most studied molecules involved in breast cancer: BRCA1 and estrogen. This is very consistent with the phenomenon being a well-known hallmark of cancer^30, 31^.

### Validation

#### Comparison with TCGA previous analyses

As our work builds on the efforts made in TCGA project, we compared our results regarding differentially expressed genes, enriched processes and network topologies with those obtained from the original papers where the samples were obtained and analysed^13, 14^.

In the seminal TCGA paper^13^, the authors analysed in an integrative fashion, mutations, mRNA and miRNA expression, methylation profiles and copy number of 825 patients, finding specific features depending on the analysed PAM50 subtype. Among the most important results reported by the TCGA consortium, we can mention the enriched processes, including the BRCA1 and BRCA2 deregulation in RB1 pathway, apoptosis evasion and proliferation, as well as, S phase and S/G2/M checkpoints. Those processes were also found enriched and corroborated by our functional analyses as depicted in Fig. 7A,B and Table 4. The original TCGA breast cancer paper also reported important mutations (amplifications, deletion and indels) in genes such as PIK3CA, PTEN, AKT1, TP53, GATA3, RUNX1 and PIK3R1. However, in our analysis PIK3R1 is the only gene that resulted differentially expressed from the genes listed above. Perhaps, this apparent discrepancy could be due to the fact that in the original TCGA paper, there was no contrast between healthy and cancerous expression levels. Nevertheless, the biological redundancy in the model under consideration is also captured in our analysis, at a functional level, by the overlap presented in the enriched processes reported, despite the discrepancy found in the DEGs and mutations. Regarding the analysis made by Ciriello *et al*.^14^, invasive lobular carcinoma (ILC) was studied by means of a multidimensional approach using 817 breast tumours classified by their histological features. Despite E-cadherin loss, significant mutations in the key regulator of the estrogen receptor, FOXA1 gene, were reported. Although, FOXA1 has been reported overexpressed in prostate cancer^32^, in our analysis it presented a log_2_FC = 1.215. But, this value was not statistically significant, according to our criteria (*FDR* < 1 × 10^−5^ and a |*log*_2_ *FC*|>1).

Finally, it is worth to mention that both previous analyses used different subclassification strategies. On the one hand, the classification used by the TCGA consortium^13^ included estrogen receptor status, PAM50 subtypes^2^ and unsupervised clustering. On the other hand, Ciriello *et al*.^14^ grouped samples according to their histological features. Conversely, the main objective of our work was to discover the most common transcriptional features in breast cancer. Hence, cancerous samples were not divided into molecular subtypes, but used as a whole and contrasted against healthy samples. After this first approach, steps towards the understanding of the unique features present in transcriptional networks for different molecular subtypes will be explored.

#### Functional analyses validation

We analysed a complementary collection of breast cancer microarray data in order to observe whether our results could be found in other data sets. For this analysis, five different experiments were used to build our comparison dataset (GSE54002^33^, GSE50567^34^, GSE42568^35^, GSE29431^36^ and GSE10810^37^), yielding a total of 641 breast cancer and 78 healthy samples. Differential expression analysis was performed using the same parameters than the TCGA dataset (*FDR* < 1 × 10^−5^ and a |*log*_2_(*fold change*)|>1), obtaining 1,546 candidate genes, with 44% of them also found in the RNA-Seq analysis (Supplementary Table S6). We also complemented our functional studies with a GeneSet Enrichment Analysis (GSEA^38^), performed to both our original RNA-Seq breast cancer data, as well as the secondary microarray dataset, to corroborate the enrichment of previous reported categories. The most enriched GSEA categories are involved in cell cycle and chromosome-related processes (Supplementary Table S7).

#### Network size effects, overfitting and results’ generality

In this work the large number of samples used, has allowed us to perform astringent cut-off analyses. However, two delicate issues could arise with a systems biology approach such as the one presented here for the transcriptional networks: i) the size effect difference between the samples used for HN and CN construction, and ii) a topology artefact in the CN due to data over-fitting.

Mutual information calculations for almost a thousand of samples is a computationally expensive task. Hence, bootstrap of smaller networks (100 samples) could take much time to have statistical significance. To overcome this challenge, we choose to divide our cancer network in parts of 110 randomly chosen samples, i.e., the same size than the healthy network, and observe whether or not the topology of those smaller networks maintained the original structure (Supplementary Figure S3). Additionally, we constructed networks with 220, 440, 550 and 660 samples, expecting to obtain the same general topology: small disconnected components which belong to the same chromosome. As we expected, the smaller networks have all the same pattern of small connected components, but more importantly, belong to the same chromosome (Supplementary Figure S4). With this calculation we show that for our work, the number of samples does not affect the results of the network. This is of the utmost importance, since the results on topology, chromosomal arrangements and functional processes involved in this analysis are neither dependent on the technology nor the sample sizes but on biological processes underlying breast cancer.

#### Hi-C validation in breast cancer cell lines

As a complementary validation strategy, we compared the interactions observed in the Healthy Network and Cancerous Network with data coming from Hi-C experiments^39^. With this technology, it is possible to perform unbiased genome-wide analysis of chromatin interactions. We compared the inter-chromosomal interactions in available data from experiments in breast cancer cell lines (MCF7) as well as non-cancer breast cell lines (MCF10a)^15^. Our data are shown in Table 5 where we compared the top 5 inter-chromosomal interactions in the healthy and cancerous networks respectively, with data from Hi-C experiments on MCF10a and MCF7 cells. Table 5 shows a strong correlation between the interactions obtained by the network approach and those obtained by experimental three-dimensional localisation of gene interactions. Furthermore, in that same paper^15^, a significant increase of inter-chromosomal associations between chr16 through chr22 in the MCF-10A genome with respect to the breast cancer MCF7 cell line is reported, in agreement with our results of loss of inter-chromosomal regulation in breast cancer. Those authors also suggest that the relative (MCF-10A/MCF-7) interaction frequency of chr18 with other small chromosomes was significantly increased in the MCF-10A cells. As it can be observed in our Fig. 3A of this paper, it is possible to observe several interactions between chromosome 18 and the rest of the genome, meanwhile in the cancerous network (Fig. 3B) these relationships disappear.

## Discussion

In this work, using 881 breast cancer whole-genome RNA-Seq samples from TCGA (780 cases and 101 controls), we analysed the structural and functional differences between these two phenotypes. We found 1,431 differentially expressed genes, from which the top overexpressed subset was associated with cell cycle, cell division and DNA repair, whereas the bottom underexpressed genes were related to motility, migration, angiogenesis and cell adhesion processes. By means of an informational theory-based network approach, we inferred and analysed transcriptional regulatory networks for RNA-Seq genome-wide breast cancer samples (CN) and compared them with non-cancer networks (HN). We observed a dramatic difference between the network topology of HN compared to the CN, mostly in the number and size of connected components at three different MI cut-off values. The HN with the middle cut-off value (0.01%), formed a giant component corresponding to the 80% of the total of genes of the network (4,214 out of 5,395), while on the contrary, the CN’s largest component only contained 134 genes. Here, for the first time, strong differences in the inter and intra-chromosomal regulation for both phenotypes are presented. Moreover, the intra-chromosomal interactions observed in the CN occur between neighbouring genes; these neighbouring regions present a consistent expression pattern: either predominantly overexpressed or predominantly underexpressed. Differences in gene expression levels between cancer and non-cancer samples, as well as between the HN and CN, were consistent with the already known hallmarks of cancer, in particular for breast cancer, such as the activity via estrogen or BRCA1 signalling pathways (Fig. 7). On the other hand, some mechanisms apparently counteract the effect of the aforementioned deregulation: the inhibition of processes related to migration or cell adhesion (Table 4, and Figs 4(A,D), 5 and 6). This approach remarks the interplay between progression and slowdown of cell communication during the development of breast cancer. In what follows, we will give a set of hypotheses partially derived from the results observed here.

### Transcription networks inference and communication loss between processes in breast cancer

A relevant matter of intense research in cancer biology is whether communication between processes is lost during cancer. Here, we built the CN and HN based on mutual information to provide a quantitative index of dependency between pairs of genes. A simple visual inspection of Fig. 1 revealed a dramatic difference in the size and number of connected components between CN and HN, i.e., a giant connected component was present in the HN meanwhile, the CN had several small components. As far as the authors know, this is the first instance of a putative loss of communication in the CN reported. Secondly, the distribution of MI values was different (See Supplementary Figure S5), even for non-differentially expressed genes. Such is the case of the most connected genes in the CN: ANKFY1 (LFC = −0.208), DPH1 (LFC = −0.1), NEURL4 (LFC = −0.274), PSMB6 (LFC = 0.094) and SLC25A11 (LFC = −0.102). Supplementary Figure S6 shows histograms of the MI values for those 5 genes in CN and HN, evidencing the same effect that is observed in Supplementary Figure S5: MI values tend to be higher in HN genes with respect to CN. This is an indicative that independently of the differential expression, the statistical dependency between healthy samples is larger than the observed between the cancerous ones. The generality of this result seems to give account to an intrinsic process of the phenomenon of cancer, more than an effect of the methodology to obtain the correlation values. We took the top 0.01% MI values to build both networks, yielding a MI cut-off value of 0.159 and 0.1745 for the CN and HN respectively. Moreover, the difference in the MI cut-off values increased by choosing larger networks. Using the strongest 0.1% MI values, i.e., one order of magnitude greater, the cut-off values would be changed to 0.068 and 0.1263 for the CN and HN respectively. Since MI provides a measure of the statistical dependence of pairs of variables which can be understood in this context as correlations in the transcriptional processes, lower levels of these values in the CN may reveal a weak co-regulation in the whole transcriptional program during cancer. At the same time, the highest outlier values of MI in the CN are close to 1 (0.986, between CKMT1A and CKMT1B), meanwhile in the HN, the highest MI value is obtained between HBA1 and HBA2 (0.687). This fact could be related to the acquirement of stronger specific relationships relevant to cancer.

In the CN it is possible to observe that all components are constituted mostly by underexpressed or overexpressed genes (Fig. 1B). Furthermore, almost all the components in the CN have at least one transcription factor (bold border of nodes), which could indicate that the regulation of those sets of genes could be governed by the interaction of that gene with the other members of the component.

To the best of our knowledge, this is the first time in which a whole-genome network analysis in cancer reports that the large majority of interactions between genes is given by the ones that belong to the same chromosome. The strongest relationships between genes in cancer at the transcriptional level are intra-chromosomal; meanwhile, in the healthy tissue, the regulation occurs between pairs of genes of any chromosome. We want to stress the marked difference in the location where the relationships occur for HN and CN, which implies dramatic differences in chromosomal regulation during the transcriptional process. We argue that this result could be due to a dysfunction in the RNA polymerase machinery.

### Deregulation of specific molecules triggers malfunction of cell cycle, migration and hormone signalling

Causal network analysis showed an important set of genes involved in cancer. Those genes are consistent with their activation state and the upregulation of carcinogenic processes, as it can be observed in Fig. 5, where the majority molecules expression values were in agreement to the exacerbated levels of cancer-related pathways, e.g. overexpression of KDM5B, an important oncogene in breast cancer^40, 41^, IQGAP3, CCNB1, CDK1 or CCNE2, which are highly involved in the correct function of cell cycle checkpoints and promotion of cell division. On the other hand, we also have two underexpressed genes such as MYC and BCL6. The MYC expression could be related to the action of BRCA1^42^. Interestingly, almost all the blue lines are directed to or from MYC/BCL6. Although they are also oncogenes, they were underexpressed, which may indicate that despite the fact that the cell is facing a strong damage, there are some mechanisms which tend to repair said damage.

Cellular component distribution of deregulated molecules also called our attention (Fig. 5). Several overexpressed molecules were found inside the nucleus whereas mostly underexpressed ones were outside of it. This observation could mean that the transcriptional process is highly active while the cell-to-cell communication is downregulated. The highest Z-scores of the Diseases and Functions corroborate the upregulation of cell division (G2 phase) in Table 4. Concomitantly, the molecules appearing in the extracellular space indicate a decrease in cell-to-cell communication supported by the bottom five Z-scores, where cell-to-cell communication is downregulated. Meanwhile, the upstream regulator analysis of COL18A1 (Fig. 6) showed that, even when this molecule is upregulated, the processes in which is involved are downregulated. Those processes are mostly related to cell adhesion and migration. It is remarkable that not one of the processes involved in the Diseases and Functions analysis with lowest Z-scores include COL18A1 (Table S3), suggesting that the downregulation of migration and cell adhesion processes might be produced by different mechanisms. This finding acquires relevance since migration and the remodelling of the extracellular matrix are classic hallmarks of cancer.

### Coexistence of mechanisms of cancer progression and DNA repair

In Fig. 7A we can observe how apparently the cell attempts to repair DNA damage, i.e., BRCA1 signalling pathway is trying to repair DNA, via the FANCD2-RAD51-BRCA1/2 complex^43^. However, E2F is also overexpressed, allowing the transcription of several other genes^44^. In the end, we may be observing, how an opposite action between a tumour suppressor gene (BRCA1) and an oncogene (E2F) takes place, during the development of breast cancer. The BRCA1/FANC/RAD51 complex is in some sense nullified and, at the same time, E2F strongly promotes G1-S progression via cyclins and CDKs (Fig. 7B). It is worth mentioning that cyclins and cyclin-dependent kinases in Fig. 7B are all overexpressed, which leads to a malfunction in the G1/S cell cycle checkpoint. In addition, there is also the interaction between c-MYC and CDC25A^45^. c-MYC is underexpressed, as we mentioned previously, possibly due to the inhibitory action of BRCA1 over the expression of c-Myc^42^. However, CDC25 manages to avoid the inhibitory effect of MYC. In this context, E2F also participates in the Estrogen-mediated S-phase entry. Interestingly, cyclins, CDKs and E2F act independently of the expression of its regulators. From the interpretation of this figure we can argue that the process of DNA synthesis is exacerbated, since several transcription factors are active. The former gives place to BRCA1 DNA-repairing signalling pathway to participate, but, the overexpression of cyclins and CDKs promotes an evasion of cell cycle checkpoints. This is a typical portrait of cancer: communication loss between processes which are collaborative working together in healthy conditions. These results led us to study the interactions in the transcriptional networks in detail.

### PCDHGs as a novel candidate to regulate cell adhesion in breast cancer

Our analyses led us to observe that PCDHGs are downregulated. Decrease in this complex inhibits cell adhesion, contraction and morphogenesis (Fig. 4B). From the network analysis, the strong association between all the elements of the complex is evident. At the same time, the enrichment analysis shows that cell adhesion processes might be downregulated by the decreased function of this complex, suggesting again that the underexpression of a given process could be obtained by different molecular mechanisms. This zone is hypermethylated in breast cancer; to direct research to understand the specific mechanisms that PCDHGs have in the context of breast cancer is appealing. It is also remarkable that all of the aforementioned processes are downregulated in the functional analysis observed in Figs 5 and 6 and Table 4.

Regarding the connectivity of the cluster, the localisation of those genes on chromosome 5 and the consistent underexpression of them, could be the reason for which they are strongly correlated in the transcriptional network. However, the mutual information approach to construct networks, is based on the expression level of the molecules. By observing Fig. 4C, it is clear that the expression level of all molecules is not exactly the same. This fact acquires relevance since is not only a conserved expression level throughout the genes, but the consistent expression pattern along the 872 samples of the breast cancer RNA-Seq samples which produces this tightly connected structure in PCDHG cluster.

### Final considerations

In this work we have performed a Systems Biology approach to unveil subtle relationships at the transcriptional level in breast cancer. To achieve this we constructed and analysed networks obtained by a theoretical information algorithm. Results obtained by this approach (differentially expressed genes, functional analysis and network topologies) were validated by independent datasets, alternative sequencing technologies and different enrichment approaches. To our knowledge this is the first time that a RNA-Seq based network inference in breast cancer reveals loss of inter-chromosomal interaction. Hi-C data also reinforce this last result, inter-chromosomal relationships are more frequent and higher in non-cancer breast cell lines (MCF10a), compared to breast cancer cells (MCF7)^15^. Furthermore, to find consistently neighbour clusters which are predominantly overexpressed or underexpressed in the cancerous network, could be indicative that the transcriptional regulation in breast cancer is highly dependent on the three-dimensional compartmentalisation. Further investigation to address this issue is necessary, but it is important to highlight that, with a coarse-grained approach it is possible to unveil geographical features that could shape the three-dimensional cellular landscape in breast cancer.

Breast cancer commonalities should be the starting point to focus efforts to discover the mechanisms underlying this disease. With this approach we have directed the research towards a global understanding of the transcriptional programs in health and disease. The approach presented here revealed that those shared features in breast cancer may provide insights regarding the acquisition or loss of specific functions that control the finely regulated transcriptional program. The heterogeneity of breast cancer is without a doubt one of the major challenges for its clinical management, and therefore it is a necessary consideration for any study of the disease. In our recent work^46^ we compared the transcriptional architecture of the commonly studied subtypes of breast cancer: luminal A, luminal B, basal, and HER2-enriched, based on microarray data, and using a similar information-theoretical strategy. In that work, we identified differences in the networks of the different molecular subtypes; however, we found the differences to be much greater between any subtype against the control samples. With this in mind, for this work we focused on the differences between tumours and regular breast tissue as the first approach using the RNA-seq technology. Considering our current findings, a logical question is whether these results are common to all molecular subtypes. We would expect to be able to explore this when a suitable large dataset is available.

Cancer has been the most important disease in the 20th century and it will also be like that for the 21st century. Understanding cancer at the molecular, proteomic, metabolic, organismal and even social level is mandatory. The inherent complexity underlying each level of description makes it virtually impossible to integrate it in a coherent fashion, given the enormous plethora of variables involved in the rise and progression of this disease. This is the principal reason to develop research focused on a specific level. Here, by inferring networks with all available next generation sequencing samples at TCGA, we clearly reveal a non-previously observed general feature in breast cancer: the loss of inter-chromosome regulation. This last addresses the problem of finding the most relevant differences between breast cancer and non-cancerous transcriptional regulatory programs. From our findings, experimental procedures to validate what we have presented here will be necessary in order to reveal to a fuller extent the mechanisms behind the appearance of this dismal disease.

## Materials and Methods

**Figure 8:**
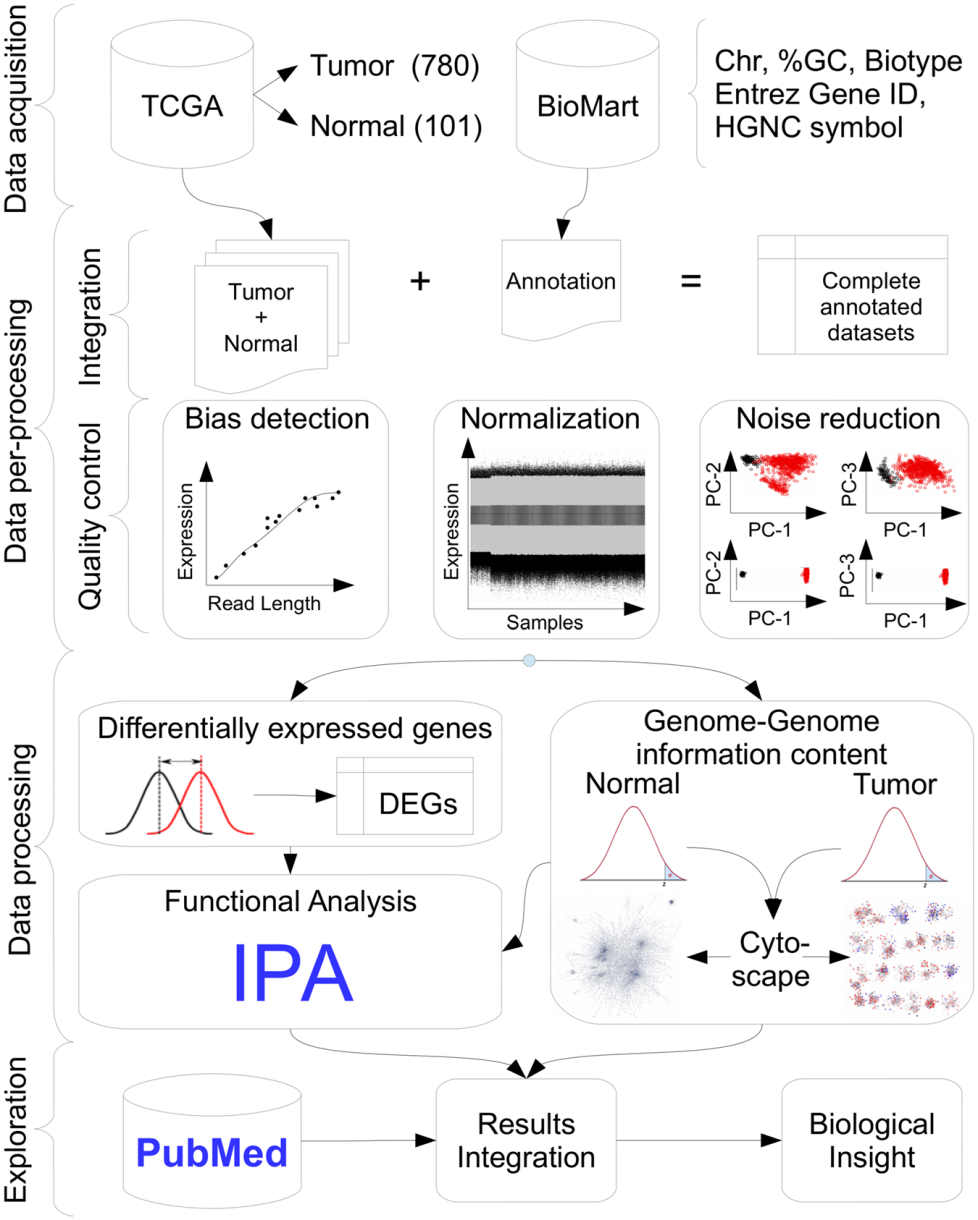
Material and methods workflow diagram. The workflow starts with the data acquisition block which gathers level three breast invasive cancer gene expression raw counts for tumour and normal datasets from The Cancer Genome Atlas (TCGA). Complementary annotation data are obtained from BioMart (Chromosome, %GC content, Entrez Gene IDs, and HUGO Gene Nomenclature Committee - HGNC - symbols). Then, the pre-processing block integrates both expression and annotation data necessary for quality control, such as bias detection (e.g. %GC content, gene length, etc.), within/between normalization and multidimensional principal component (PC) analysis noise reduction. After that, the processing block diverges into two complementary analyses: i) Differentially expressed genes (DEGs) discovery is carried out to find potential candidate genes between cases and controls; ii) Two whole genome-genome mutual information density distributions are built for each condition in order to construct similar networks and visualized with Cytoscape. Both DEGs and networks results are then submitted to functional analysis with Ingenuity Pathway Analysis (IPA). Finally, result exploration integrates PubMed literature together with functional results to obtain biological insight of the problem at hand.

The present work includes four processing data blocks (acquisition, pre-processing, processing and result exploration) which are described in detail below and depicted in the workflow diagram of Fig. 8. All the statistical analyses for this article were done using R version 3.2.2^47^.

### Data Acquisition

This whole work is based upon data generated by the TCGA Research Network^48^ (http://cancergenome.nih.gov/). All available breast invasive carcinoma datasets were downloaded, restricted to level three data from UNC (IlluminaHiSeq_RNA-Seq) platform with matching tumour and normal samples up to July 2015, yielding a total of 780 and 101 files respectively (Supplementary Table S3). The starting point considered in this work were the 20,532 raw counts obtained at the gene expression level with corresponding Entrez Gene ID^49^ and symbol provided by the HUGO Gene Nomenclature Committee (HGNC)^50^ for each sample.

Complementary annotation data were obtained from BioMart^51^ using Ensembl Genes 80 database for *Homo sapiens* (GRCh38.p2), in order to obtain the following fields: Chromosome name, gene start and end, %GC content, gene/biotype (protein coding, snoRNA, lincRNA, snRNA, etc.), Entrez Gene ID, HUGO Gene Nomenclature Committee (HGNC) symbol and HGNC ID(s).

### Data Pre-processing

This block can be conceptually divided into two: i) Integration and ii) Quality control as detailed described below.

#### Integration

Basically, integrity check had to be carried out in raw expression files to control that all of them have both the same dimension and provided TCGA identifiers before complementary annotation can be incorporated. In this context, the following filtering criteria were applied to fulfil this task:

**BioMart filter:** Only records with complete Entrez Gene ID and Symbols fields, belonging to conventional chromosomes (1, 2… 22, X and Y) were kept.

**Data merge:** The Entrez Gene ID was used as a primary key to join the expression and annotation data. If more than one BioMart candidate records were found, both TCGA and HGNC symbols had to match. If additional records were found the one with lowest GC content was selected.

The above criteria resulted in a 19,449 × (780 + 101|10) expression matrix, where genes are in rows and samples (tumour and normal) plus 10 complementary annotation entries are in columns.

#### Quality control

NOISeq R library was used for global quality control in order to assess several aspects (See NOISeq quality control report in Supplementary Material)^52, 53^. First, the relative biotype abundance in the experimental conditions were evaluated in order to assess if samples contained protein coding expression genes in their majority as confirmed by the Supplementary Material results. Second, gene counts expression boxplots were evaluated per biotype to confirm that the highest median expression corresponded to protein coding genes. Third, saturation plots were obtained, i. e., the number of detected genes (counts >0) per sample across different sequencing depths as simulated by NOISeq.

All samples reached saturation for the number of detected features at the corresponding sequencing depth, i. e., no further gene will be detected. Fourth, global expression quantification for each experimental condition yielded a feature sensitivity >60% for 10 count per million (CPM), which suggest an accurate library preparation. Fifth, different bias detection plots were tested, where bins containing the same number of corresponding ordered genes based on their mean gene length, %GC and RNA content were plotted against their corresponding mean expression of gene counts. Unfortunately, the three tested bias presented a pattern and should be removed in order to avoid inappropriate biological conclusions.

EDASeq R library was used for batch effect removal^54^. Before normalization genes with mean counts <10 were filtered resulting in 17,215 genes, as suggested in ref. 54. Different within/between normalization strategies were tested to remove bias presence (See Supplementary Table S8). The best alternative was sequentially full quantile GC content and gene length within normalization followed by Trimmed Mean of M values (TMM)^55^ between normalization. Within full quantile normalization consisted in matching the distribution of the gene counts to a reference distribution defined in terms of median counts across the artifact to be removed (%GC or gene length) for each sample. Between normalization using TMM assumes that the majority of the genes is not differentially expressed and empirically equates the overall gene expression levels between samples based on a reference sample. The TMM scaling factor value for each sample is a weighted sum of the log-fold change of each gene with respect to the reference sample, with weights as the inverse of its approximate asymptotic variance. However, this sum is trimmed in the sense that it uses only the genes that were not present in the lower and upper 30%/5% of log-fold change and average expression values respectively. Afterwards, NOISeq analysis confirmed artifacts removal (See Supplementary Figure S7).

Sample *log*_2_(normalized count) expression densities exploration showed a consistent bi-modal pattern, corresponding to noisy lower expressed genes and global sample behaviour. Filtering out features with low counts (*CPM* <10 cut-off) retained 15,281 genes removing the undesired lower density peak (See Supplementary Figure S8). Multidimensional sample exploration based on Principal Component Analysis (PCA) scatter scores plots, showed that experimental group overlap exists and each condition presented different variance. ARSyN R library was used for multidimensional noise reduction using default parameters (See Supplementary Figure S9) ^56^. This strategy is useful to remove systematic noise and/or batch effects. Basically, it decomposes the expression matrix into a sum of matrices according to an analysis of variance (ANOVA) using the experimental design plus the random error term. Then, each matrix can be analysed by PCA and reconstructed using only the first eigenvalues that explain more that 75% of the data, plus its appropriate error. The filtered expression matrix would result from subtracting to the original expression matrix, the error of each factor matrix reconstruction plus the signal of the random error term.

### Data processing

This block diverges into two complementary analysis: i) Genome-wide interaction network analysis for each experimental condition and ii) Differential expressed genes (DEGs) between normal and tumour conditions. Then, both DEGs and networks results were submitted for functional analysis.

#### Network construction

Several correlation measures have been used to develop transcriptional interaction networks based on the inference of statistical dependency^57–60^. It has long been proven that the best estimator of statistical dependency is mutual information (MI)^60–62^. The Algorithm for the Reconstruction of Accurate Cellular Networks (ARACNE)^61, 62^ is a well-known information-theoretic algorithm that correlates pairs of genes by MI values^62^. In order to make comparable both networks, we kept only the top 0.01% MI values for healthy networks (HN) and cancerous networks (CN), yielding 11,675 interactions. The lowest MI values were 0.174 and 0.159 for the HN and CN respectively. Interestingly, tumour density shifted to a lower narrow MI compared to the normal case (see Supplementary Figure S3). To study the networks’ topological properties, we use the network analyzer plug-in of Cytoscape (v.3.2.1). For this work we focused on degree centrality measures, number of connected components, i.e. groups of nodes connected between them, but not connected to the rest of the network and size of these components.

To further explore that the generality of the network’s results and connectivity characteristics are not caused either by overfitting nor by different sample sizes (110 and 780 for healthy and cancerous respectively), a 7-fold validation strategy over the cancerous samples was carried out using the same MI cut-off value. In addition, cancerous networks with healthy sample size x {2, 3, …, 6} were also tested. No difference in topological characteristics nor overfitting bias effect was present in both cases, i.e., results were similar for every tested complementary cancerous trained network.

#### Differentially Expressed Genes

Independent gene-based linear models were adjusted using limma R package^47^ to find DEGs in tumour samples compared to the healthy samples using (1):

1 $${y}_{ij}={\mu }_{+}{\alpha }_{i}+{\varepsilon }_{j}$$

where *y*_*ij*_, is the *log*_2_(*normalizedgeneexpression*); *μ*, is the global mean; *α*_*i*_ is the i-th experimental condition (normal or tumour) and *ε*_*j*_ *N* (0, *σ*) is the random error term of the j-th sample. Hypothesis tests based on empirical Bayes moderation of the standard errors towards a common value, were used to obtain the corresponding p-values which were adjusted to control multiple comparisons using the False Discovery Rate (FDR)^63^. Due to the fact that each experimental condition has a large number of samples, model (1) had a strong statistical power. Thus, the DEGs were defined as the **1,431 (9.36%)** genes that had a *FDR* < 1 × 10^−5^ and a |*log*_2_(*fold change*)| > 1 in order to find those differentially expressed genes with statistical significance, where the random number of expected genes is 0.15281. Visual inspection confirmed that these DEGs could accurately separate the experimental conditions in a heatmap and represent a manageable number for further functional analysis and complementary biological validation. Complete model results of the differential expression analysis can be found in Supplementary Table S2.

#### Complementary microarray dataset

For comparison purposes, data from breast cancer samples analysed on the Affymetrix HGU133plus2 platform (GPL570) were collected from the Gene Expression Omnibus (GSE54002^33^, GSE50567^34^, GSE42568^35^, GSE29431^36^ and GSE10810^37^). Microarray data were processed following a pipeline using the Robust Multi-array Average^64^, with batch effects controlled with the ComBat algorithm^65^.

#### Functional analysis

Gene expression analysis often falls short in our attempts to gain biological insight about complex, heterogeneous phenotypes such as cancer, due to the very large number of differentially expressed genes commonly displaying inconsistent behavior among samples. In order to increase the predictive capacities derived from high-throughput omic experiments, a number of methods commonly termed Pathway Analysis have been developed recently. One important approach to pathway analysis of biomolecular data is the application of statistical data mining techniques in highly curated databases. One of such databases, perhaps the more comprehensive to date, is QIAGEN’s Ingenuity Pathway Analysis (IPA, QIAGEN Redwood City, www.qiagen.com/ingenuity), which has been developed, compiled and continually curated by scientists at Qiagen.

Our DEGs were then submitted to functional analysis with IPA. We generated causal networks through this platform. This uses a highly curated knowledge-based source: the Ingenuity Knowledge Base (IKB). It contains more than 40,000 nodes representing mammalian genes and their products (transcripts, proteins, miRNAs) as well as 1,480,000 interactions between them. The aforementioned links represent experimentally observed cause-effect relationships relating to transcription, expression, activation, molecular modification, etc. Based on this information, IPA contains several Canonical Pathways constructed according to molecules which participate in specific processes in the cell. Diseases and function and Upstream regulator categories are another kind of molecule sets which are available with this tool. For further methodological details please see ref. 66. Causal network analysis is a valuable tool to find the common pathways in specific categories of interest such as cell signalling processes in cancer. IPA allows for us to find directed relationships between our DEGs and those whose relationships are over-represented in a given canonical process.

Enrichment scores of gene expression experimental data within the IKB framework are determined by hypergeometric tests or Fisher exact tests –depending on the statistical dependency conditions on the variables under consideration– that measure the overlap between observed and predicted gene sets. Z-score analyses are used to assess the match between observed and predicted up/down regulation patterns allowing for Bayesian scoring of the results.

As a secondary analysis, Gene Set Enrichment Analysis^38^ was performed. For this analysis, the complete collection of Canonical Pathways available from the Molecular Signature Database was used, considering all genes measured in the RNASeq experiments, as well as the microarray data.

## Electronic supplementary material

Supplementary Information

Dataset 1

Dataset 2

Dataset 3

Dataset 4

Dataset 5

Dataset 6

Dataset 7

Dataset 8
