# Supplementary material for: RNA-Seq based genome-wide analysis reveals loss of inter-chromosomal regulation in breast cancer
Source: Sci Rep. 2017 May 11;7:1760. doi: 10.1038/s41598-017-01314-1 (PMC5431987; doi:10.1038/s41598-017-01314-1)

RNA-Seq based genome-wide analysis reveals loss of inter-chromosomal regulation in breast cancer

Jesús Espinal-Enríquez[1,2,+], Cristóbal Fresno[3+], Guillermo de Anda-Jauregui[1], and
Enrique Hernandez-Lemus[1,2,*]

1Computational Genomics Division, National Institute of Genomic Medicine (INMEGEN), 14610, México
2Centro de Ciencias de la Complejidad, Universidad Nacional Autónoma de México (UNAM), 04510 México
2UA AREA CS. AGR. ING. BIO Y S, CONICET - Universidad Católica de Córdoba, Argentina
*ehernandez@inmegen.gob.mx
+these authors contributed equally to this work

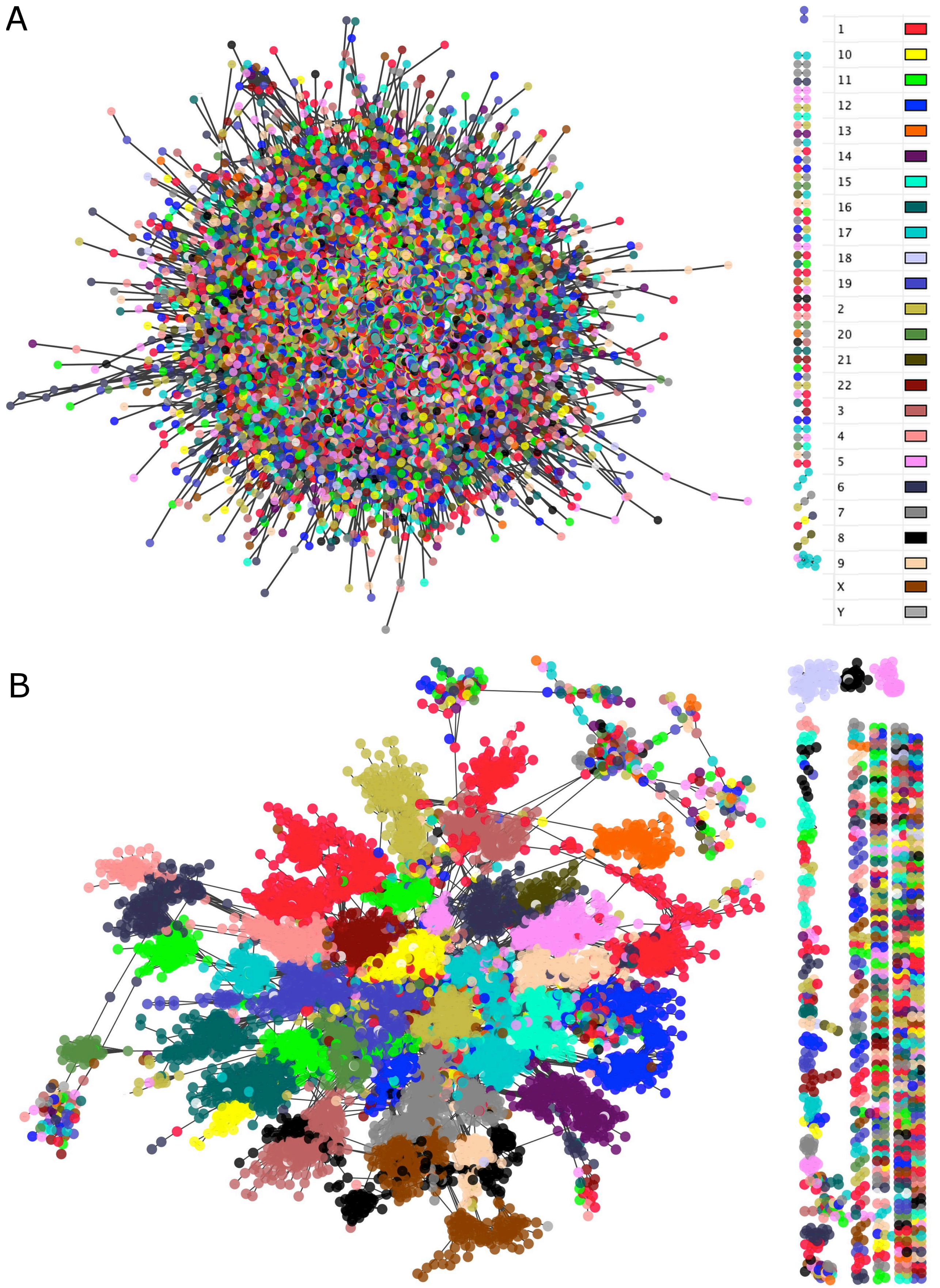

Fig. S1. Networks at 0.1% of mutual information value. A) Healthy network. B) Cancerous network (CN). Both networks are depicted with the same layout as in Fig 6. The colour code is according to the chromosome location in which each gene is located. Despite the fact that both networks have a giant component, a simple visual inspection shows that the CN's gene are more connected to other genes of the same chromosome than to other chromosomes.

A

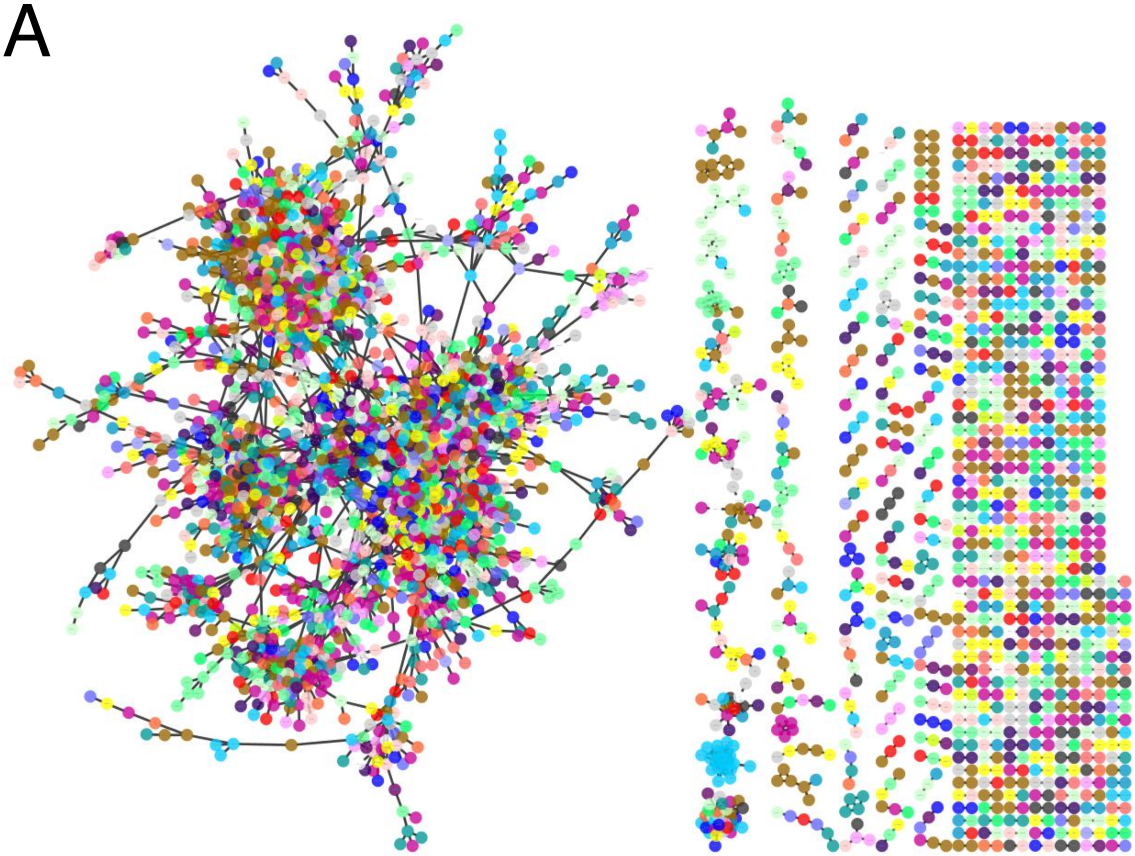

B

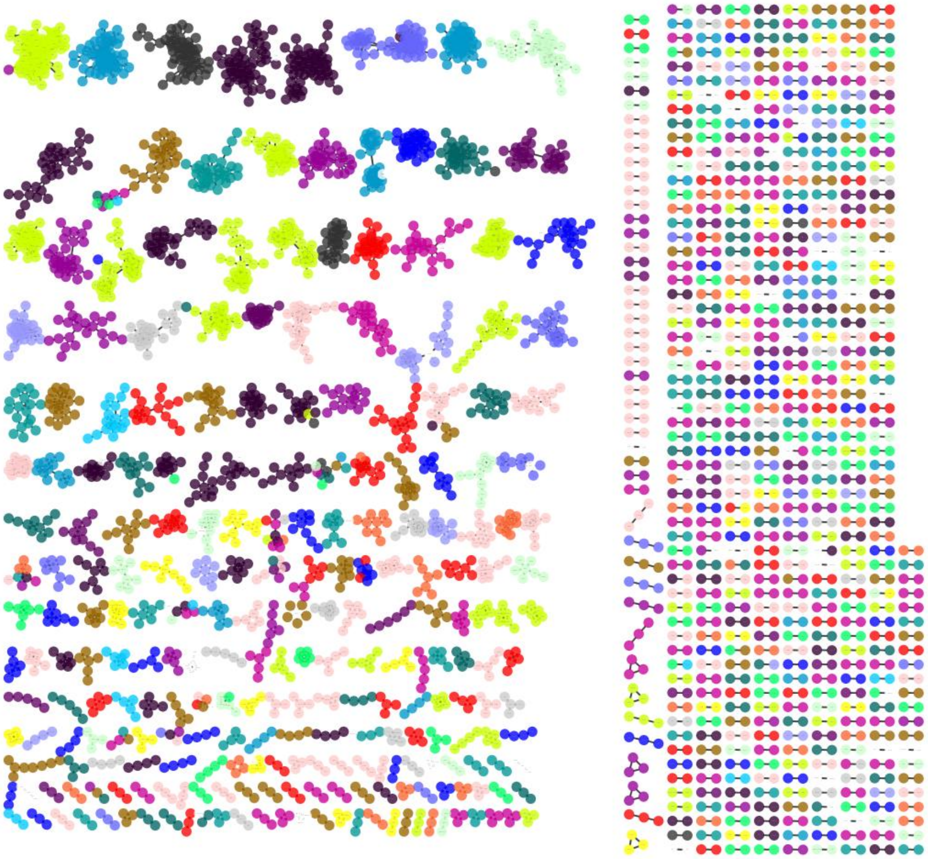

Fig. S2. Networks at 0.001% of mutual information value. Healthy network (HN) B) Cancerous network (CN). Both networks are depicted with the same layout as in Fig 6. The colour code is according to the chromosome location in which each gene is located. Notice that gene connections are similar to the ones showed in Fig. 6.

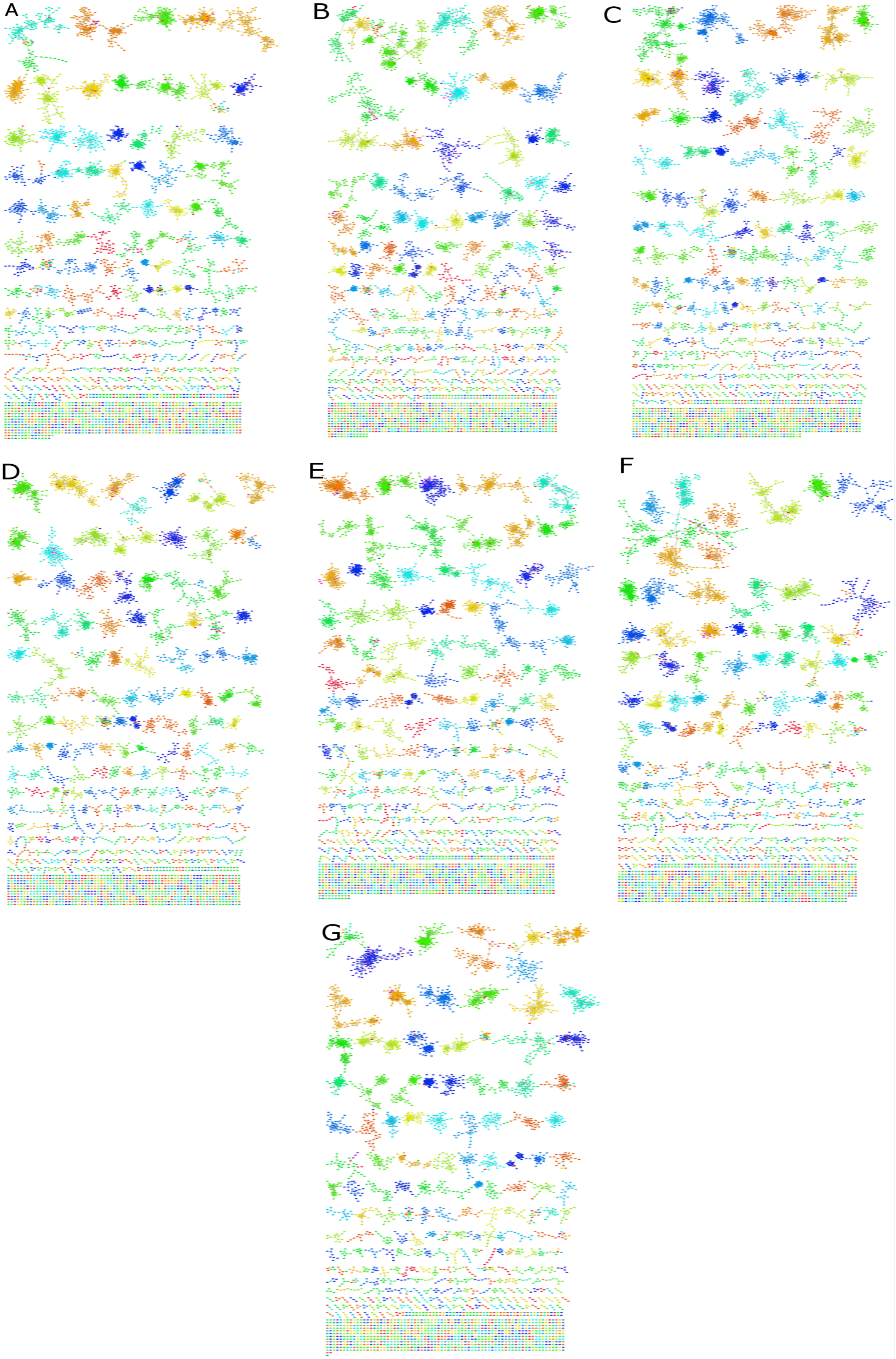

Fig. S3. Network structure does not depend on the number of samples. Networks were constructed with 110 random samplings. A) to G). Genes are coloured according to the chromosomal location of each gene. Notice that for each network, the structure and connectivity are maintained: loss of inter-chromosomal regulation and small connected components.

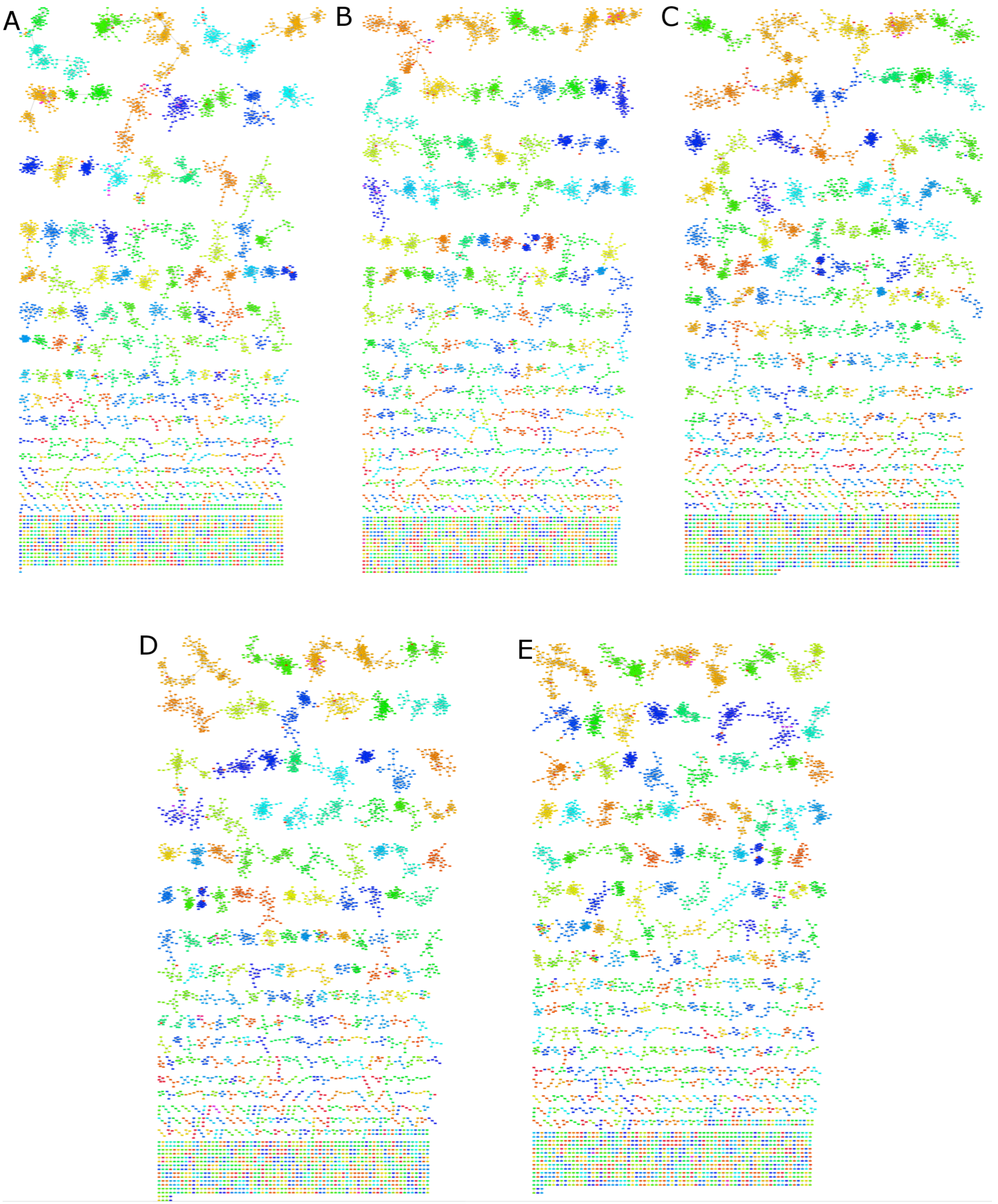

Fig. S4. Validation cancerous networks with larger number of samples. To corroborate the number of samples does not determine the network structure. Networks were constructed with A) 220 random samplings. B) 330 random samplings C) 440 random samplings D) 550 random samplings and E) 660 random samplings. Genes are again coloured according to the chromosomal location of each gene.

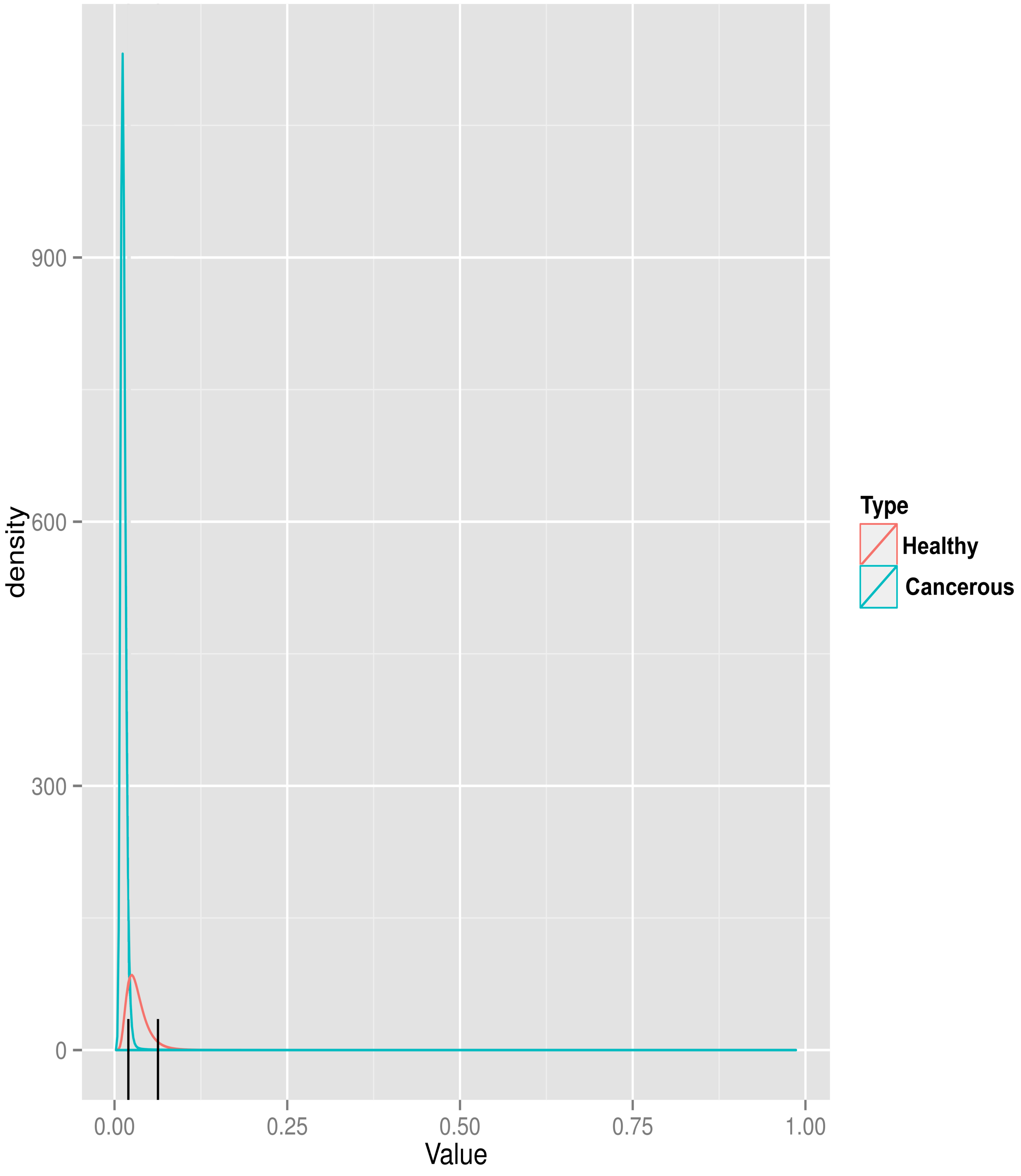

Fig. S5. Mutual information density plots. Healthy and cancerous density plots are shown in red and cyan colour respectively. Vertical black lines represent the top 0.01% cut-off for each experimental condition. Note the density shift to lower mutual information values for cancerous condition compared to the healthy case.

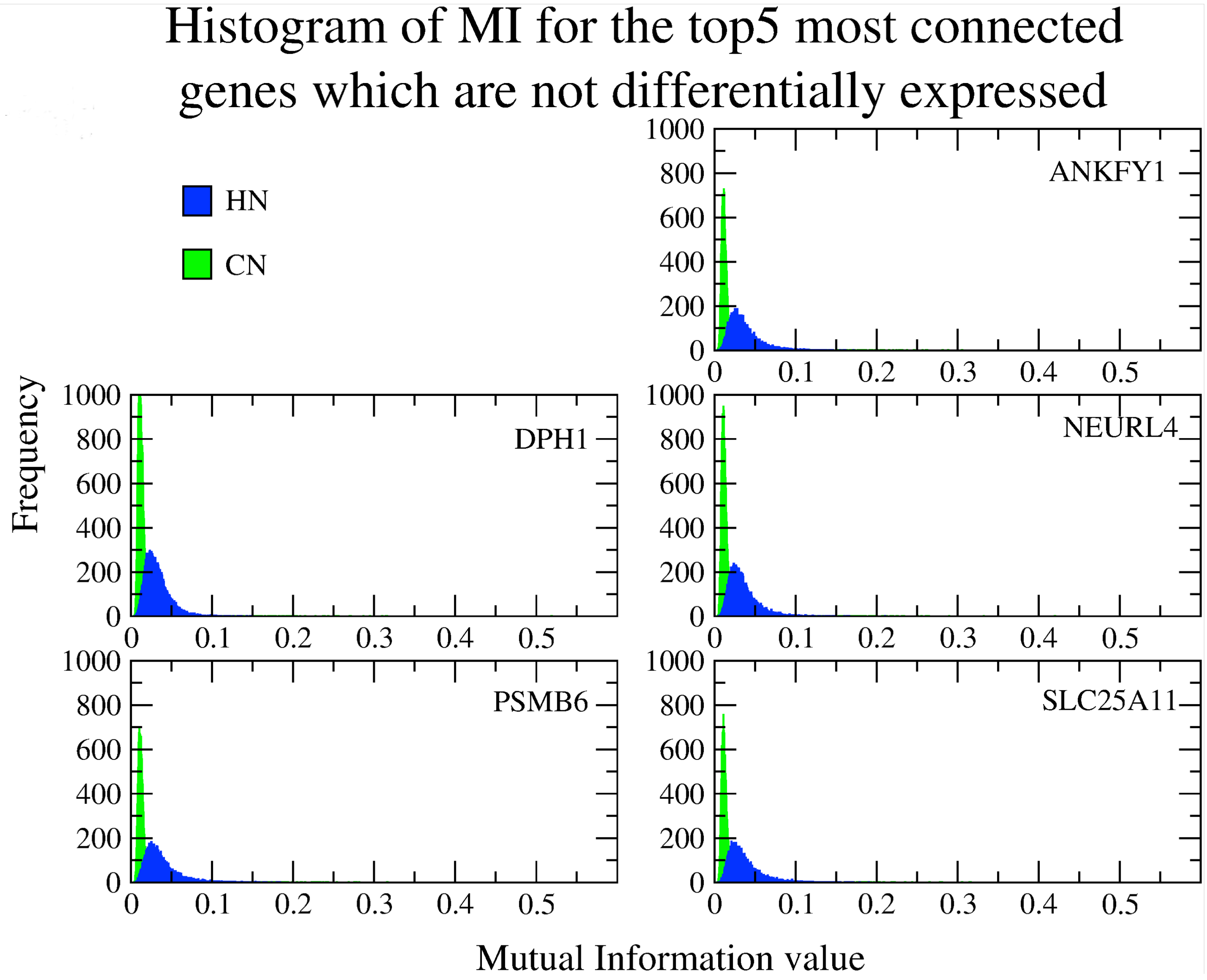

Fig. S6. Histogram of Mutual Information for the 5 most connected genes in the CN and they are not differentially expressed. Each plot represents the frequency of MI values between the mentioned genes and the rest of the genome. Each histogram contains 15,642 values of MI. In blue, the distribution of Healthy samples are depicted, meanwhile for cancerous network, the distributions are in green. Notice that blue values tend to be higher than the green ones, as it can be expected from the visual inspection of Fig. S5.

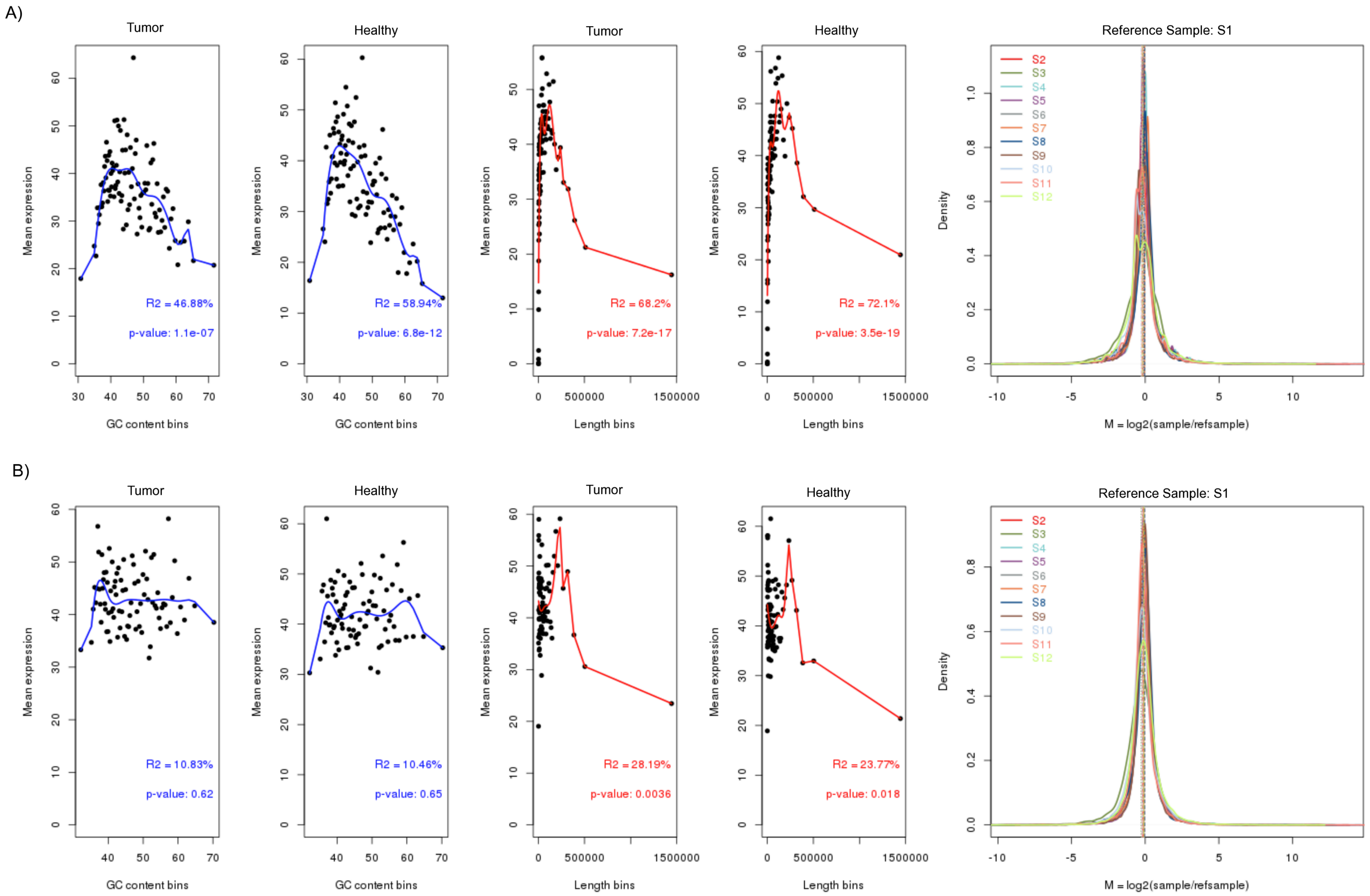

Fig. S7. Artefact removal assessment. A) Original NOISEq GC content (blue), gene length (red) and RNA content bias detection plots for cancerous and healthy experimental conditions. For the first four scatter bias plots the X axis represents the centre value of 200 features bins for the respective artefact against its 5% trimmed gene mean counts, which is then fitted with a cubic spline regression model (coloured line). The RNA content panel shows the density of the M=log2(sample/reference simple) for each sample. In this case sample one (S1) was taken as reference by default. B) Similar plots obtained after EDASeq within and between normalisation for bias removal.

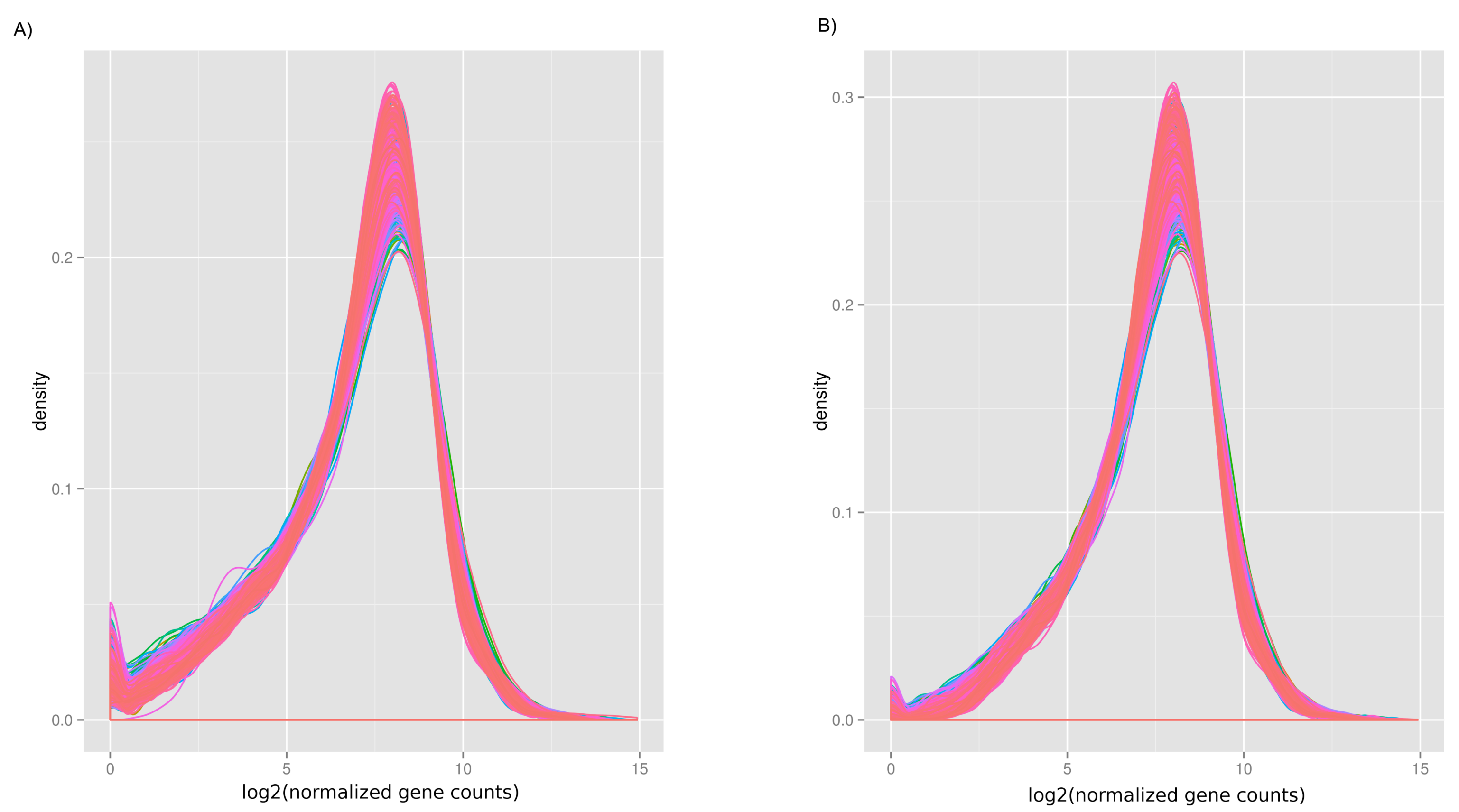

Fig. S8. Normalized samples expression density plot. Values are expressed in log2(normalized genes counts) scale. A) Original bimodal plot. B) Lower expressed genes artefact were removed after CPM < 10 filter was applied.

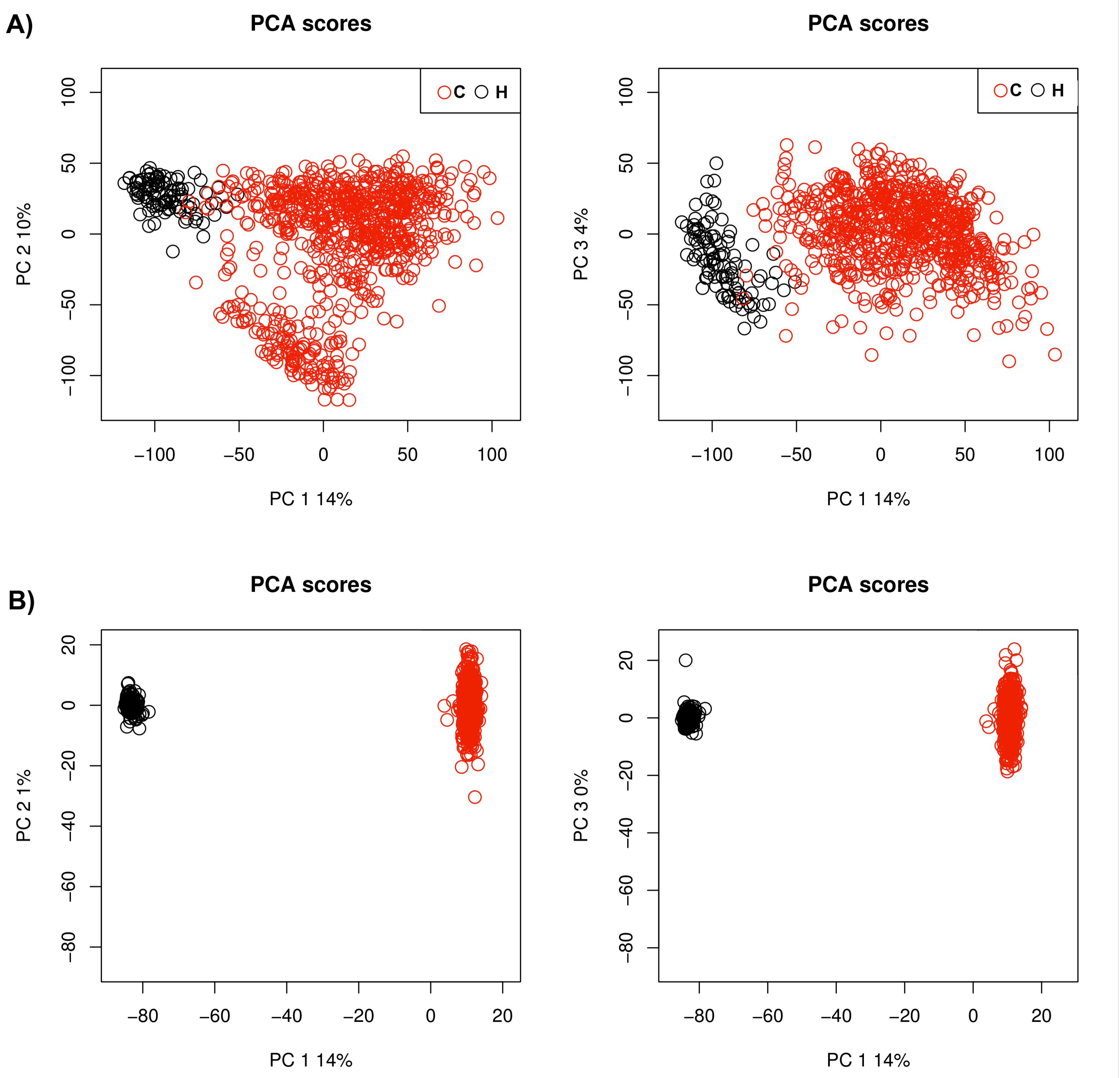

Fig. S9. Multidimensional noise reduction. A) Original scatter principal component (PC) analysis score plot for the first-second/third components for healthy (black) and cancerous (red) samples. B) Similar plot after ARSyN noise reduction

Quality Control of Expression Data

Generated by NOISeq on 08 Jul 2015, 12:42:05

Content

Plot	*Description*
**Biotype detection**	Biotype abundance in the genome with %genes detected (counts > 0) in the sample/condition.<br>Biotype abundance within the sample/condition.
**Biotype expression**	Distribution of gene counts per million per biotype in sample/condition (only genes with counts > 0).
**Saturation**	Number of detected genes (counts > 0) per sample across different sequencing depths
**Expression boxplot**	Distribution of gene counts per million (all biotypes) in each sample/condition
**Expression barplot**	Percentage of genes with >0, >1, >2, >5 or >10 counts per million in each sample/condition.
**Length bias**	Mean gene expression per each length bin. Fitted curve and diagnostic test.
**GC content bias**	Mean gene expression per each GC content bin. Fitted curve and diagnostic test.
**RNA composition bias**	Density plots of log fold changes (M) between pairs of samples.<br>Confidence intervals for the median of M values.

Biotype detection

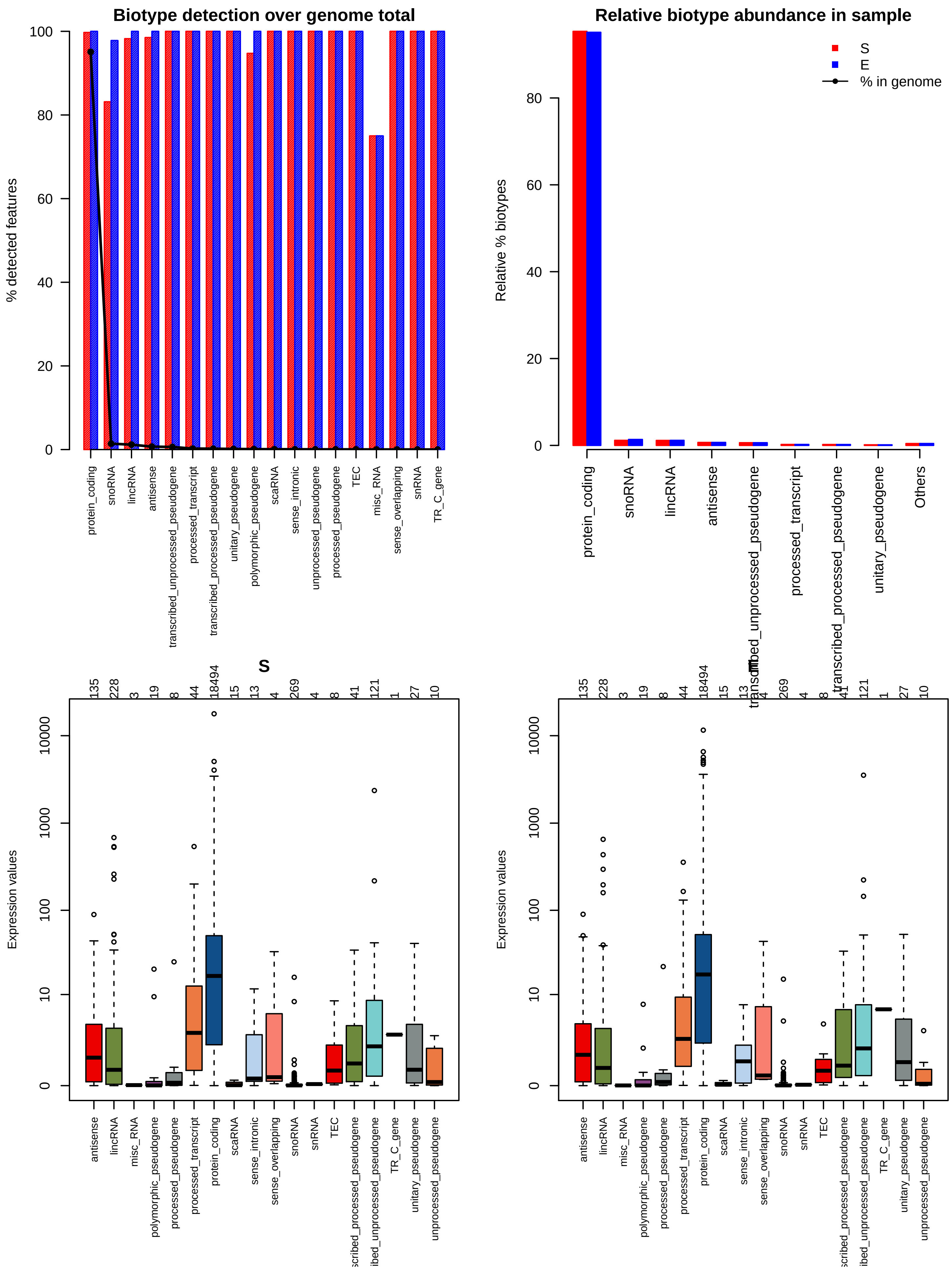

Sequencing depth & Expression quantification

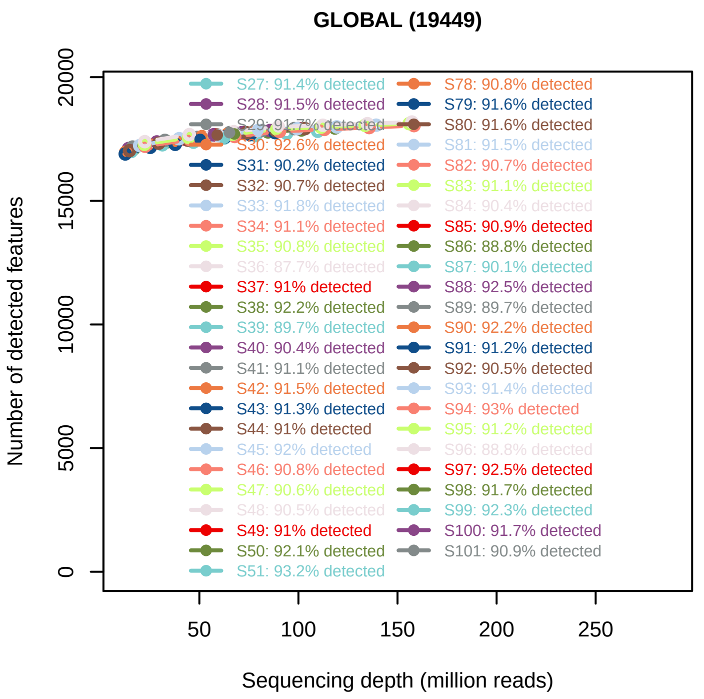

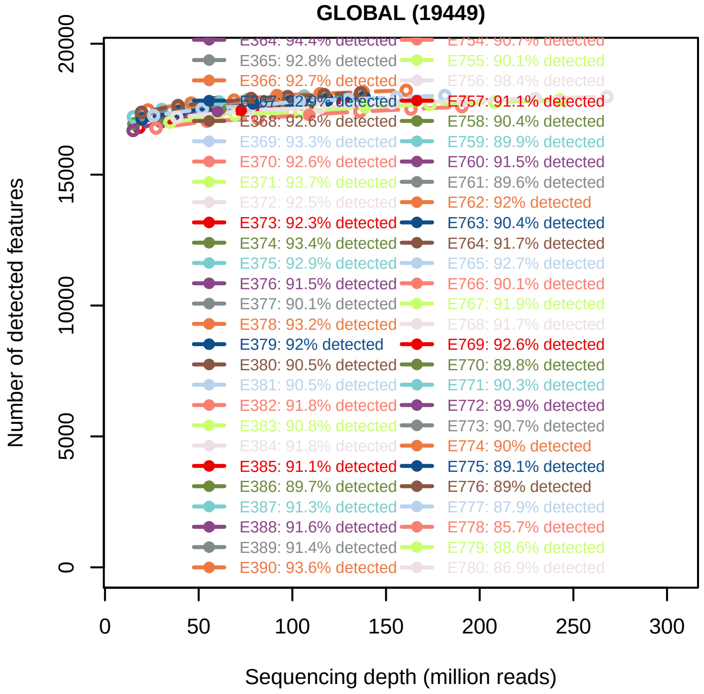

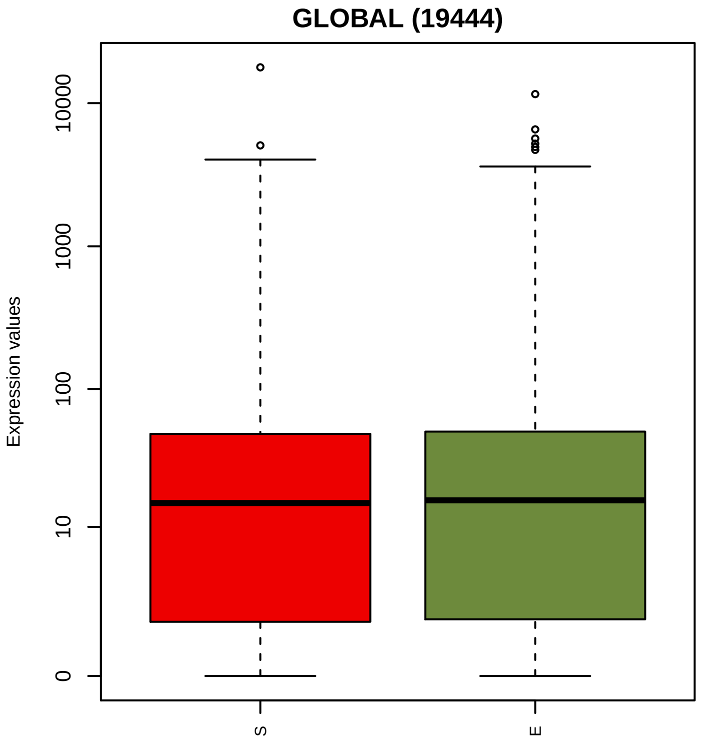

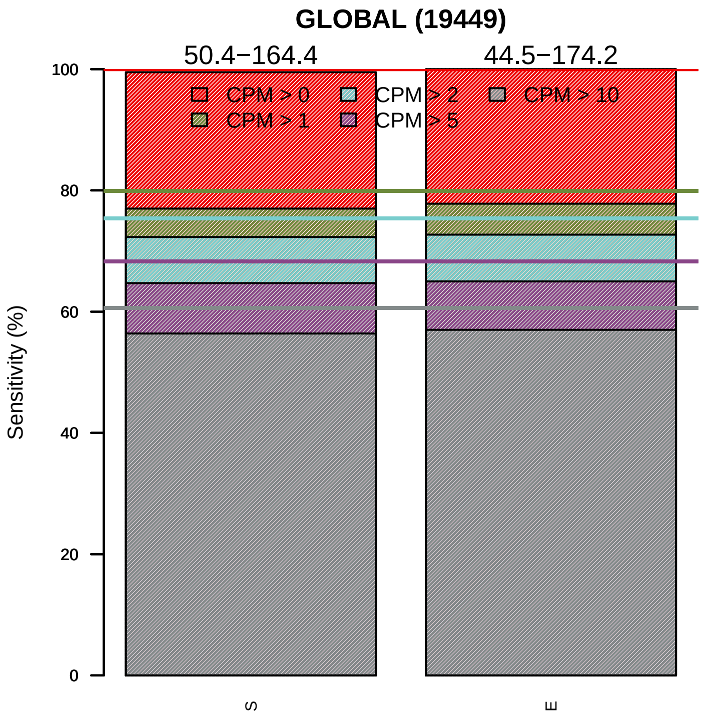

Sequencing bias detection

Diagnostic plot for feature length bias

FAILED. At least one of the model p−values was lower than 0.05 and R2 > 70%.

Normalization for correcting length bias is recommended.

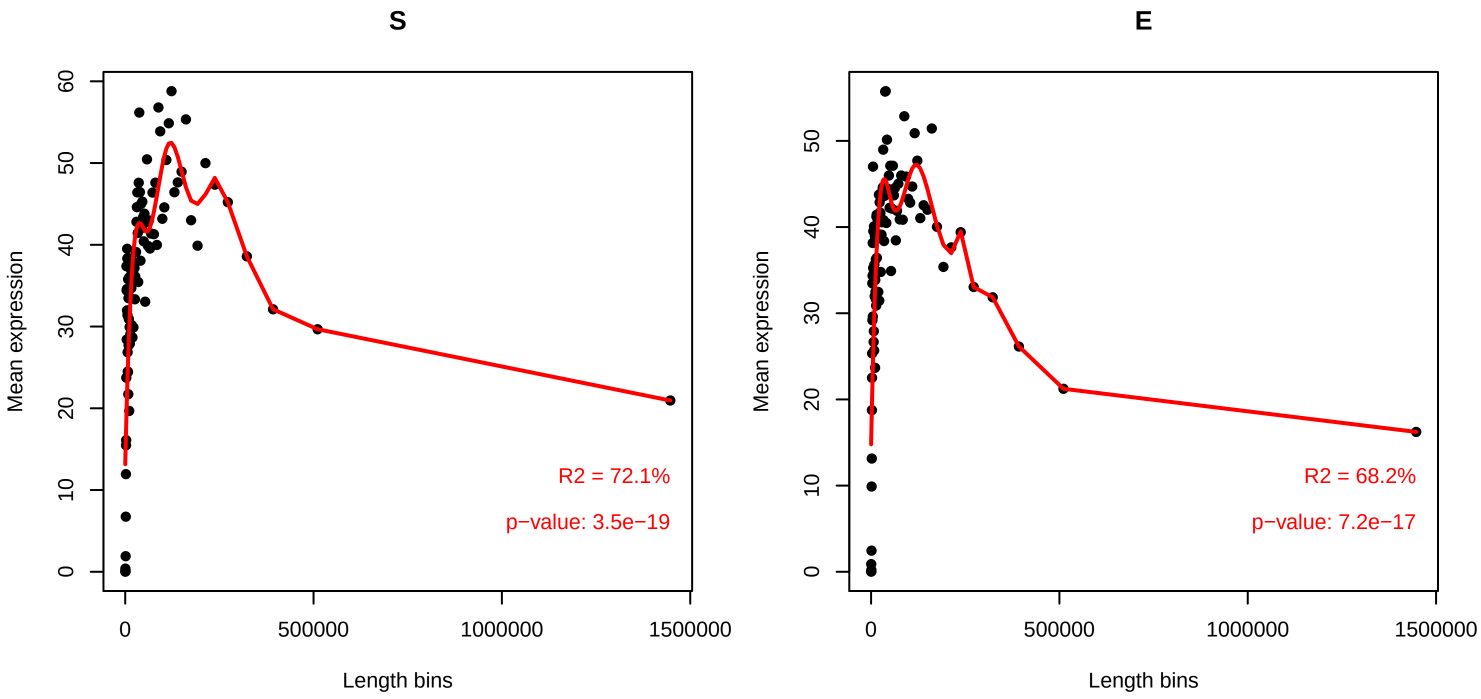

Diagnostic plot for GC content bias

WARNING. At least one of the model p−values was lower than 0.05, but R2 < 70% for at least one condition.

Normalization for correcting GC content bias could be advisable.
Plese check in the plots below the strength of the relationship between GC content and expression.

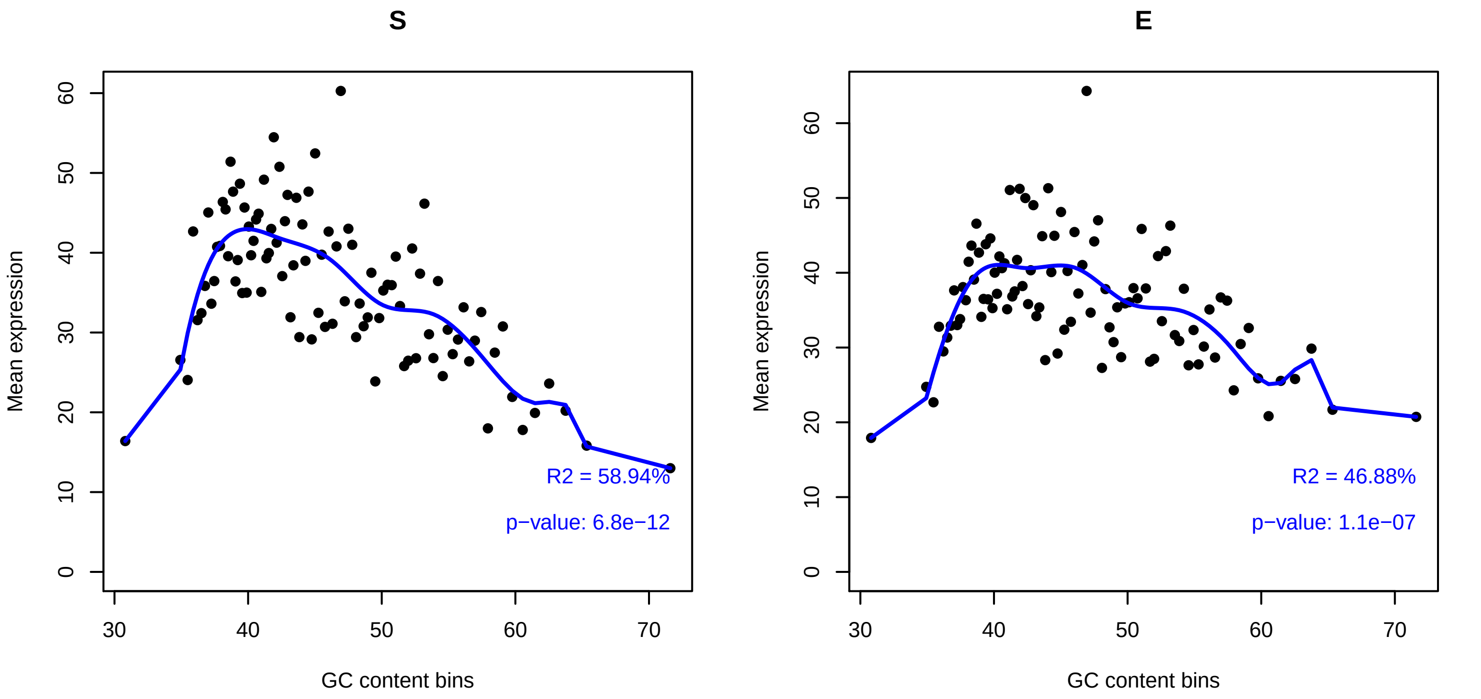

Diagnostic plot for differences in RNA composition

FAILED. There is a pair of samples with significantly different RNA composition

Normalization for correcting this bias is required.

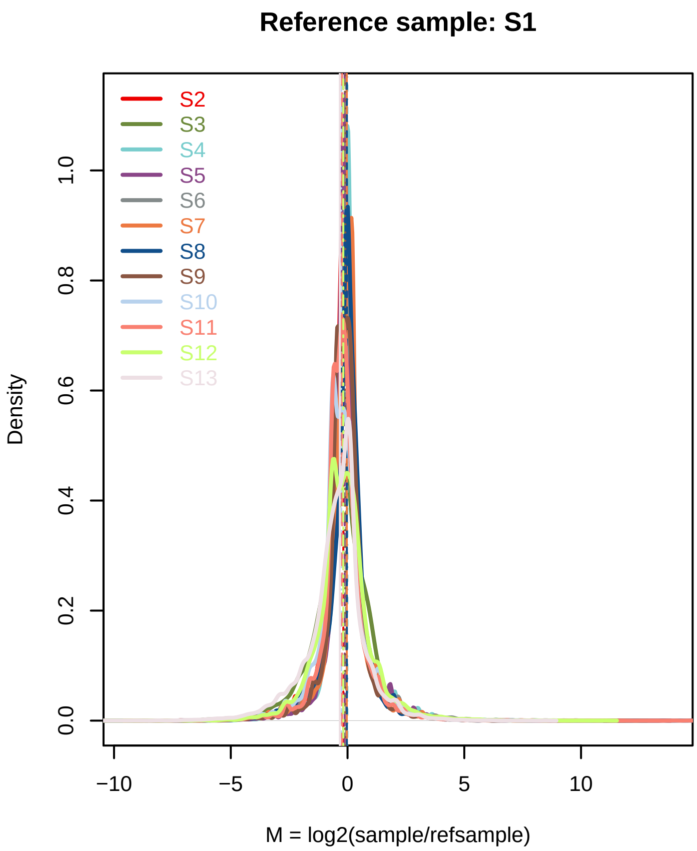

Confidence intervals for median of M values

Sample	0%	100%	Diagnostic Test
S2	−0.2	−0.1609	FAILED
S3	−0.1265	−0.1163	FAILED
S4	−0.0189	0.0061	PASSED
S5	−0.1375	−0.1086	FAILED
S6	−0.0816	−0.0438	FAILED
S7	−0.0486	−0.0148	FAILED
S8	−0.0293	−0.0293	FAILED
S9	−0.1098	−0.0766	FAILED
S10	−0.2892	−0.2455	FAILED
S11	−0.2529	−0.2177	FAILED
S12	−0.2044	−0.1587	FAILED
S13	−0.3625	−0.3003	FAILED
S14	0.0015	0.0244	FAILED
S15	−0.1161	−0.0895	FAILED
S16	−0.5055	−0.5055	FAILED
S17	−0.2871	−0.246	FAILED
S18	−0.2087	−0.1655	FAILED
S19	−0.1765	−0.1439	FAILED
S20	0.0103	0.0298	FAILED
S21	−0.114	−0.0774	FAILED
S22	0.0039	0.0251	FAILED
S23	−0.069	−0.0374	FAILED
S24	0.0603	0.0603	FAILED
S25	−0.568	−0.4871	FAILED
S26	−0.0908	−0.0572	FAILED
S27	−0.1499	−0.1234	FAILED
S28	−0.096	−0.0667	FAILED
S29	−0.123	−0.0969	FAILED

Exploratory PCA

Use this plot to see if samples are clustered according to the experimental design.

Use ARSyNseq function to correct potential batch effects.

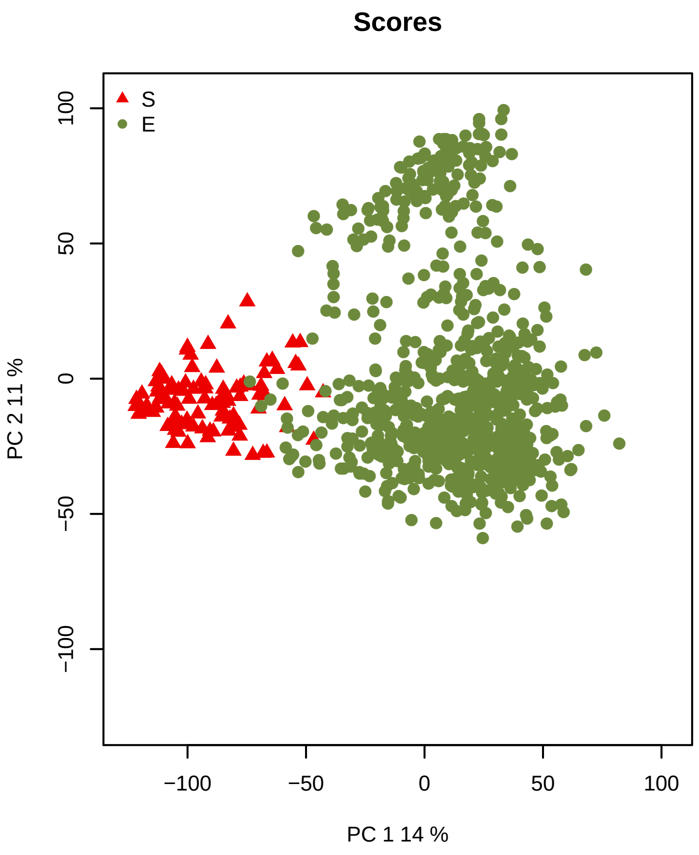

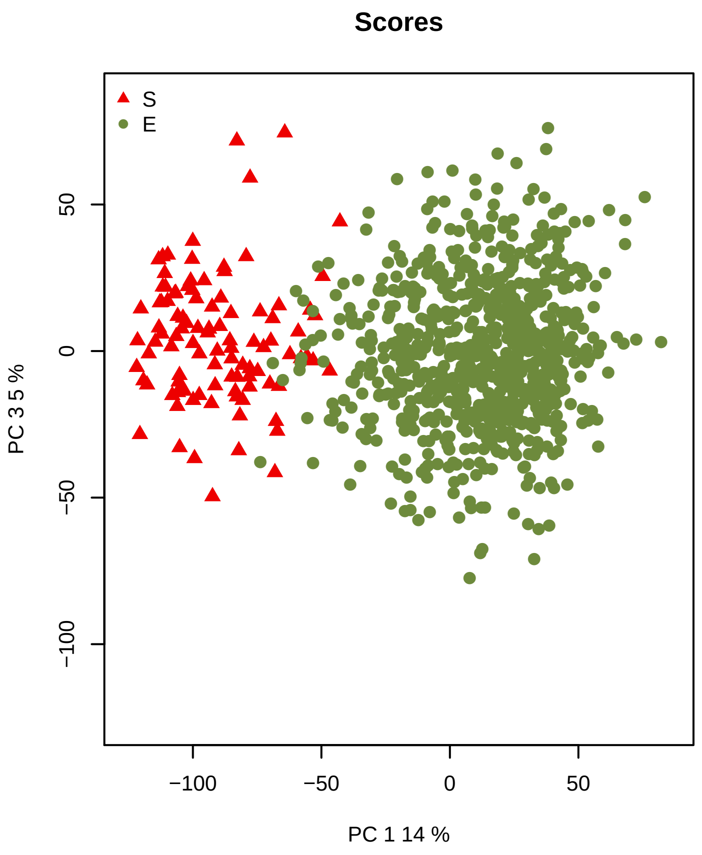

Supplement: Supplementary file 1 — Supplementary Information [file 41598_2017_1314_MOESM1_ESM.pdf]
